# “Looking up” linked to feeling down: a meta-analysis of online upward social comparison and psychological maladjustment

**DOI:** 10.3389/fpsyg.2026.1825169

**Published:** 2026-05-15

**Authors:** Yuqing Lei, Shirui Hu, Yidan Sun, Lijun Zheng

**Affiliations:** 1 Faculty of Psychology, Southwest University, Chongqing, China; 2 School of Psychology, Shanxi Normal University, Taiyuan, China

**Keywords:** digital distress, psychological maladjustment, social media, social-evaluative negative emotions, upward social comparison

## Abstract

**Introduction:**

Social media has become a routine setting for self-evaluation, and upward social comparison with perceived better-off others may be associated with psychological maladjustment. However, the average magnitude of this association across outcome domains and the contextual factors that may moderate it remain unclear.

**Methods:**

This meta-analysis synthesized 94 effect sizes from 54 independent samples (*N* = 36,583) using a three-level random-effects framework that modeled dependence among effect sizes within studies.

**Results:**

The estimated average correlation between upward social comparison and psychological maladjustment was $\bar{r}$ = 0.330, 95% CI [0.289, 0.370]. Outcome domains differed significantly, *QM*(4) = 47.84, *p* < 0.001. After reverse-coding positively valenced outcomes, the largest average estimate was observed for social-evaluative negative emotions ($\bar{r}$ = 0.438), followed by anxiety ($\bar{r}$ = 0.382), depression ($\bar{r}$ = 0.306), lower wellbeing ($\bar{r}$ = 0.268), and lower self-esteem ($\bar{r}$ = 0.263). No significant moderating effects were detected for age, cultural background, or data collection year in the outcome-specific models, and exploratory design-based comparisons did not indicate stronger overall effects in longitudinal than in cross-sectional studies.

**Discussion:**

Online upward social comparison is, on average, associated with multiple indicators of poorer psychological functioning, with particularly strong links to social-evaluative negative emotions. These findings highlight the need for future longitudinal and culturally contextualized research on digital comparison processes.

## 1 Introduction

Social media has become deeply embedded in everyday life. As of 2024, global social media users have surpassed 5 billion, representing over 62% of the world's population, with average daily use exceeding 2 h and 23 min (Kemp, 2024). These figures suggest that social media is not merely a leisure activity but a routine social environment in which people evaluate themselves in relation to others. Unlike offline settings, where individuals are more likely to encounter a fuller picture of others' lives, social media platforms disproportionately present polished and favorable content. Users are therefore repeatedly exposed to others' achievements, appearance, and lifestyles, a pattern that can intensify upward social comparison—the tendency to compare oneself with others perceived as better off (Festinger, 1954; Steers et al., 2014; Vogel et al., 2014).

These concerns are also reflected in public health reports. The World Health Organization has noted a sharp rise in problematic social media use among adolescents (World Health Organization, 2024). Recent epidemiological data likewise indicate increases in internalizing problems among youth, with the incidence of clinical depression and anxiety rising by over 50% and 30%, respectively, over the past 5 years (Xiang et al., 2024). This pattern—greater connectivity alongside poorer psychological wellbeing—has intensified interest in the possible costs of digital engagement. Although early theoretical perspectives such as the stimulation hypothesis suggested that exposure to successful others might encourage self-improvement, a growing body of research indicates that, for many users, “looking up” online is associated with psychological maladjustment (White and Lehman, 2005; Schmuck et al., 2019; McComb et al., 2023). As smartphones make these comparisons increasingly routine, the gap between the self and idealized online others may become a recurrent source of distress, often described as “looking up and feeling down” (Schmuck et al., 2019; Hjetland et al., 2024). Rather than being confined to a single emotional state, these adverse associations appear to span multiple domains of mental health.

### 1.1 The spectrum of psychological consequences

While adverse effects of upward social comparison are widespread, existing syntheses often treat its diverse consequences as parallel endpoints, overlooking the distinct underlying psychological processes. Drawing on serial mediation frameworks, it has been posited that proximal, specific emotional reactions often precede distal, global wellbeing decrements (Li, 2024). Consequently, a “proximal-distal” framework is adopted here to organize the scope of existing research by distinguishing between immediate, acute responses and cumulative mental health states. We note that this framework is used as an organizing heuristic for grouping outcome categories, not as a causal model that the present meta-analysis can directly test.

The first domain, social-evaluative negative emotions, represents the proximal and acute reactions. This category specifically encompasses envy, body dissatisfaction, fear of negative evaluation, and loneliness (Xiang and Kong, 2024). Drawing on Social Self-Preservation Theory (SSPT), these specific emotions are classified as proximal outcomes because they represent distinct social-evaluative reactions (Dickerson and Kemeny, 2004). This theory posits that humans possess a fundamental psychobiological system dedicated to preserving the “social self,” defined as one's social status and acceptance. When this system detects a threat to social standing, such as being outperformed by a superior peer, it preferentially triggers specific self-conscious emotions like envy, shame, and humiliation rather than generalized distress.

Unlike general anxiety or depression, these emotions serve the functional purpose of signaling a direct threat to one's social rank. Specifically, seeing others' success may trigger envy as a signal of status deficit, while exposure to idealized bodies may induce body dissatisfaction as a marker of physical inadequacy (Li, 2024; Xiang and Kong, 2024). Furthermore, the fear of negative evaluation reflects an anticipatory anxiety about losing social approval (Tong et al., 2017). These are acute, immediate affective responses theoretically linked to the interpersonal contrast.

In contrast to these proximal reactions, the second domain encompasses generalized mental health indicators, representing more distal consequences of comparison. While the social self-preservation system is designed for acute signaling, chronic activation of this system may exert a sustained impact on generalized mental health indicators. This includes contributing to depression and anxiety while being associated with diminished wellbeing (Appel et al., 2016; Verduyn et al., 2020). The mechanism is theorized to operate through a cumulative process of self-devaluation. Constant exposure to the idealized highlight reels of others may create a persistent discrepancy that gradually erodes the user's self-esteem (Vogel et al., 2014). Recent research indicates that upward comparison mediates the link between passive usage and diminished social self-efficacy (Mo and Peng, 2023). Over time, this chronic mental strain may solidify into more stable symptoms, manifesting as heightened anxiety about one's status and depressive cognitions regarding one's perceived inferiority (Li, 2019; Berkout and Flynn, 2025).

### 1.2 Moderators: contextualizing the effects

Although prior research generally links upward comparison to psychological distress, the reported strength of this relationship varies across studies, suggesting that contextual factors likely play a role. The magnitude of the associations between upward comparison and psychological outcomes may vary as a function of cultural background, historical context, and developmental stage, informed by both theoretical mechanisms and empirical inconsistencies observed in the field.

Cultural background serves as a critical yet often overlooked moderator. While foundational studies have predominantly focused on Western populations, a growing body of research from Eastern contexts suggests potential variability in how comparison is appraised (Vogel et al., 2014; Wang et al., 2023; Zuo and Zan, 2025). Based on Self-Construal Theory, individuals in Western cultures characterized by an independent self may perceive superior others as a direct threat to their unique competence (Markus and Kitayama, 1991). In contrast, the interdependent self-construal prevalent in Eastern cultures might buffer this psychological threat through a focus on relational harmony, thereby attenuating the link between upward comparison and self-worth (White and Lehman, 2005).

Furthermore, the impact of online comparison may vary across both historical and developmental contexts. Regarding the temporal dimension, the literature spans a decade of rapid technological change, transitioning from the text-heavy interfaces of the 2010s to the hyper-visual, algorithmically curated feeds of the 2020s (Liu et al., 2017; Jia, 2025; Zuo and Zan, 2025). This shift toward visual-centric affordances may intensify the vividness and frequency of exposure to idealized imagery, raising the question of whether the magnitude of the association between comparison and distress has changed over time (Fardouly and Vartanian, 2016).

Concurrent with these environmental shifts, distinct developmental considerations associated with age also require clarification. Empirical findings are currently mixed. While some studies identify adolescents as a high-risk group due to the fluidity of identity formation and sensitivity to imaginary audiences, others report comparably strong associations among young adults facing pressures regarding career and relationships (Niu et al., 2016; Schmuck et al., 2019; Burnell et al., 2024; Jin et al., 2024). From a neurodevelopmental perspective, the restructuring of the social brain during adolescence creates heightened sensitivity to peer feedback, yet it remains an open question whether this susceptibility diminishes or merely shifts in focus as individuals mature into adulthood (Steinberg, 2017).

### 1.3 The present study

Quantitative research in digital mental health has progressed from general usage metrics to the examination of specific psychological pathways. Some meta-analyses primarily examined the frequency of engagement, while others identified isolated risk factors, such as the link between passive usage and envy or the impact of appearance-focused interactions on body image (Appel et al., 2016; Liu and Baumeister, 2016; Saiphoo and Vahedi, 2019; Yoon et al., 2019). Although recent reviews have made strides in consolidating these findings, there remains a need to further clarify the structural relationships among these psychological outcomes (McComb et al., 2023). Furthermore, considering the rapid proliferation of social media functionalities and the exponential growth of related literature in recent years, an updated, systematic synthesis is essential to capture the current state of evidence in the field.

Accordingly, the present study provides an integrated quantitative summary of the average magnitude of associations between upward social comparison on social media and a range of psychological maladjustment indicators. We use a three-level random-effects meta-analytic framework that retains all eligible effect sizes from each study while explicitly modeling within-study and between-study heterogeneity. Building on this synthesis, we organize the diverse outcomes identified in prior research and examine whether the magnitude of these associations varies as a function of cultural background, data collection year, and developmental stage. We also recognize that the psychological correlates of digital distress may differ across outcome domains. Some theoretical perspectives suggest that social-evaluative emotions may be more directly tied to comparison than generalized symptoms such as depression (Li, 2024; Xiang and Kong, 2024). Examining differences across outcome categories is therefore informative for understanding the structure of these associations. We use the “proximal-distal” framework as an organizational heuristic: it groups outcomes that are theoretically more immediate (e.g., envy, body dissatisfaction) and outcomes that are theoretically more cumulative (e.g., depression, reduced wellbeing), but the present meta-analysis estimates moderation rather than mediation or temporal precedence.

By synthesizing data from 54 independent samples involving 36,583 participants, we aim to provide a nuanced quantitative picture of how upward comparison on social media is associated with mental health indicators. Guided by this objective, we propose the following hypotheses:

**Hypothesis 1:** On average, upward social comparison will show a significant negative association with self-esteem and wellbeing, and a significant positive association with anxiety, depression, and social-evaluative negative emotions.
**Hypothesis 2:** The magnitude of these average associations will vary as a function of cultural background, age group, and data collection year.
**Hypothesis 3:** Average effect sizes will differ across outcome categories, with social-evaluative negative emotions expected to show numerically larger estimates than more generalized outcome domains.

## 2 Methods

This meta-analysis was conducted in accordance with the Preferred Reporting Items for Systematic Reviews and Meta-Analyses (PRISMA) guidelines. The following sections detail the literature search strategy, study selection process, data extraction, and analytic procedures.

### 2.1 Literature search and inclusion criteria

To identify relevant studies examining the relationship between social media upward comparison and psychological outcomes, a comprehensive systematic literature search was conducted across seven electronic databases (from January 2006 to November 2025): Web of Science, PsycINFO, PubMed, EBSCOhost (Academic Search Complete), Springer Link, ScienceDirect, and Wiley Online Library. The search strategy employed a precise combination of keywords and Boolean operators to capture the intersection of social comparison, digital platforms, and mental health. Specifically, the following search string was applied across all databases, adapted to each database's syntax where necessary: (“upward social compar^*^” OR “upward compar^*^” OR “comparison with better-off others”) AND (“social media” OR “social network^*^” OR “Instagram” OR “Facebook” OR “TikTok” OR “Weibo”) AND (“anxi^*^” OR “psychological distress” OR “worr^*^” OR “depression” OR “self-esteem” OR “self-worth” OR “wellbeing” OR “wellbeing” OR “life satisfaction” OR “happiness” OR “envy” OR “jealousy” OR “body dissatisfaction” OR “body image” OR “loneliness” OR “fear of negative evaluation” OR “shame”). To minimize language bias, no language restrictions were imposed during the electronic search. In addition to the electronic database search, the reference lists of identified articles and relevant review papers were manually scanned to ensure the inclusion of all eligible studies, particularly those reporting on outcome domains not fully captured by the electronic search terms.

Studies were included in the meta-analysis if they met the following criteria:

(1) Employed quantitative research designs, including cross-sectional, longitudinal, or experimental methods, whereas those relying solely on qualitative methods, single-case designs, or theoretical reviews were excluded.
(2) Specifically assessed exposure to upward comparison targets on social media—defined as comparing oneself to others perceived as better-off—while research focusing on general social comparison frequency or offline interactions was deemed ineligible.
(3) Measured at least one of the targeted psychological outcomes, such as anxiety, depression, wellbeing, self-esteem, or social-evaluative negative emotions.
(4) Appeared in peer-reviewed academic journals to ensure methodological rigor, thereby excluding non-peer-reviewed sources such as dissertations, conference abstracts, or editorials.
(5) Provided either a directly reported zero-order correlation or sufficient statistical information corresponding to the target bivariate association between upward social comparison and a focal psychological outcome to permit conversion to Pearson's r. Conversions were retained only when the reported statistic clearly indexed the focal zero-order association and sufficient information was available for transparent conversion. Studies were excluded when the available statistics reflected only multivariable-adjusted estimates, did not correspond to the target bivariate association, or could not be converted without imposing strong additional assumptions.

### 2.2 Study selection

**Figure 1:**
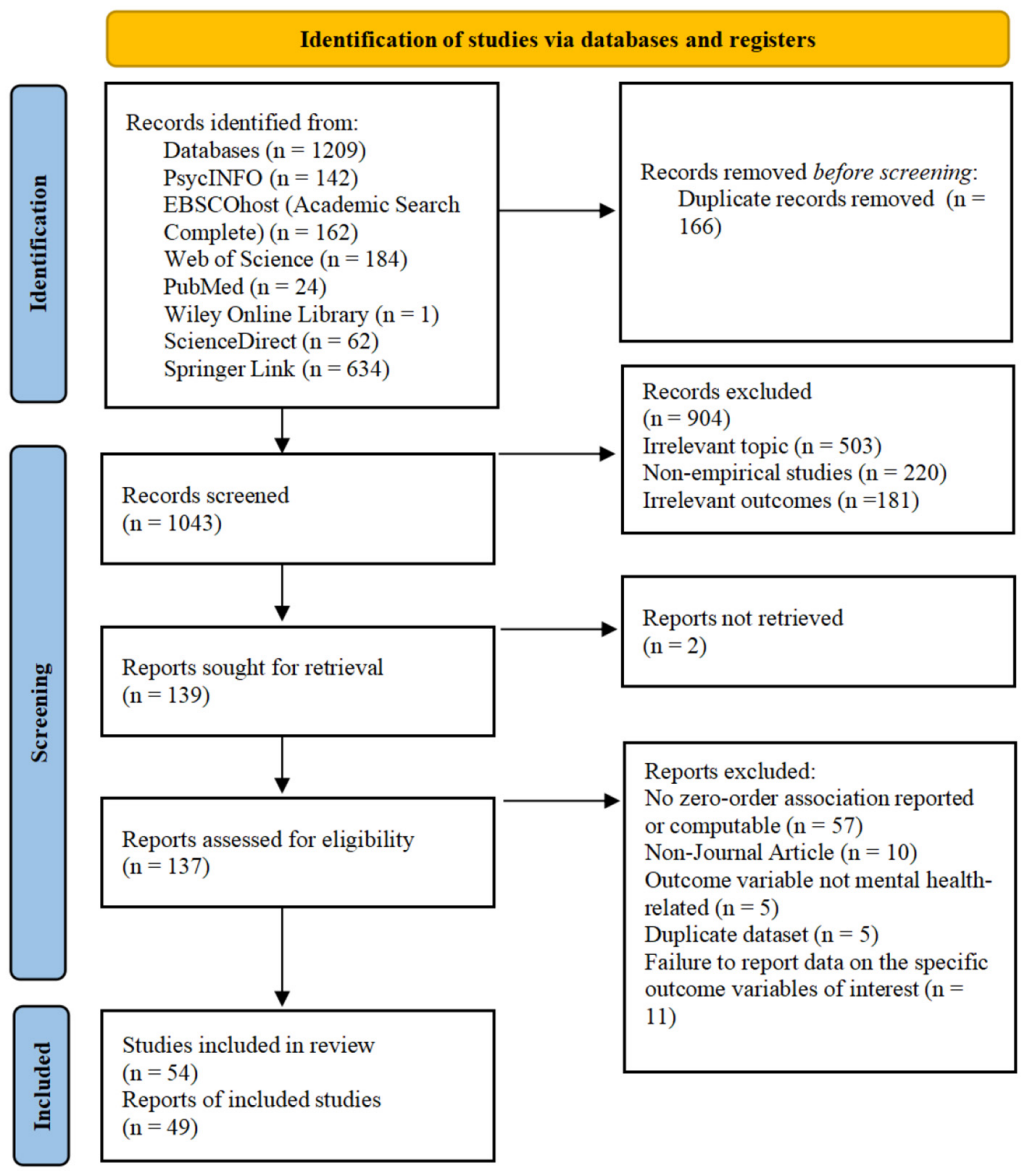
Flow chart of the study selection process for the meta-analysis.

The study selection process is illustrated in the PRISMA flow diagram (see Figure 1). A systematic search across seven electronic databases (PsycINFO, EBSCOhost Academic Search Complete, Web of Science, PubMed, Wiley Online Library, ScienceDirect, and Springer Link), supplemented by manual reference-list screening, yielded 1,209 records in total. After importing the references into EndNote reference management software, 166 duplicates were removed, leaving 1,043 unique records. Titles and abstracts of these records were screened for relevance independently by two authors, resulting in the exclusion of 904 records (irrelevant topic, *n* = 503; non-empirical studies, *n* = 220; irrelevant outcomes, *n* = 181). Of the remaining 139 reports sought for retrieval, full texts could not be obtained for 2. The remaining 137 reports were assessed for eligibility by the same two reviewers, with disagreements resolved through discussion with a third researcher. During this stage, 88 reports were excluded for the following reasons: they did not report a zero-order association between upward social comparison and a target outcome, nor did they provide sufficient statistical information from which that association could be validly converted to Pearson's r. In most of these cases, only multivariable-adjusted estimates were reported, the available statistic did not correspond clearly to the focal predictor–outcome pairing, or the information required for conversion was incomplete (*n* = 57). The remaining reports were excluded because they were non-journal articles (*n* = 10), involved outcome variables that were not mental-health related (*n* = 5), used duplicate datasets (*n* = 5), or failed to report data on the specific outcome variables of interest (*n* = 11).

### 2.3 Type of dependent variables

The outcome measures of interest were categorized into five distinct groups based on the constructs they assessed:

(1) Anxiety: Included measures of state and trait anxiety, as well as social anxiety specifically related to social media use.
(2) Depression: Comprised measures assessing depressive symptoms and depressive moods.
(3) WellBeing: Encompassed indicators of wellbeing, specifically life satisfaction, positive affect, and general happiness.
(4) Self-Esteem: Included global measures of self-esteem and self-worth.
(5) Social-Evaluative Negative Emotions: Aggregated outcomes reflecting socially-oriented or self-conscious distress, namely body dissatisfaction, loneliness, envy, and fear of negative evaluation.

Here, the grouping refers to conceptual categorization rather than statistical aggregation into a single composite. Specifically, life satisfaction was classified under WellBeing rather than as a separate happiness category; general happiness and positive affect were coded in the same domain because they all indexed positively valenced psychological functioning. Measures of self-worth were coded under Self-Esteem. Outcomes such as envy, body dissatisfaction, loneliness, and fear of negative evaluation were coded under Social-Evaluative Negative Emotions. When a study reported multiple conceptually distinct indicators, these were retained as separate effect sizes and modeled as dependent observations within the three-level framework rather than averaged into a composite score.

### 2.4 Meta-analytic data extraction

The following information was extracted from each eligible study: study characteristics (author, publication year, and data collection year); sample demographics (sample size and mean age); cultural background (operationalized as Western or Eastern based on the country of data collection); and the precise measurement tools used for upward social comparison and psychological outcomes. Pearson's correlation coefficients (*r*) were used as the effect size metric, and sample sizes were recorded to determine the statistical weight of each effect size in the meta-analysis. When a correlation coefficient was not directly reported, all full-text reports were re-screened to determine whether alternative statistics (e.g., beta coefficients, odds ratios/risk ratios, log odds, *t* statistics, *F* statistics, chi-square values, means/SD/N, and Cohen's d) could be converted to *r* for the focal zero-order association. Statistics were converted only when they corresponded clearly to the target zero-order association between upward social comparison and the focal psychological outcome and could be transformed using established formulas without requiring strong additional assumptions. Statistics were not converted when they reflected only multivariable-adjusted models, when they could not be unambiguously linked to the focal predictor-outcome association, or when the information required for transparent conversion was incomplete. This approach was adopted to avoid mixing adjusted and unadjusted effect sizes and to reduce distortion arising from tenuous conversions. In practice, only a very small number of otherwise eligible reports met these criteria; in the present dataset, one study in the depression domain operationalized the outcome as a binary diagnosis of major depressive disorder, and its reported odds ratio was converted to a correlation effect size for inclusion in the meta-analysis. To preserve all available information and properly account for non-independence, each effect size was retained as a separate observation, with statistical dependence among effect sizes from the same study handled through the three-level model described below. When a single report contained multiple independent samples, each sample was treated as a distinct Level-3 cluster. To ensure the accuracy and consistency of the data, data extraction was performed by the first author and independently cross-checked by a second author; any discrepancies were resolved through discussion with a third researcher. A PICOS-structured table of study characteristics (Supplementary Table 1) and the complete reference list of included studies are provided in the Supplementary material.

### 2.5 Moderator coding

Three pre-specified moderators were coded based on theoretical relevance. Age was coded as a categorical variable, classifying samples as either adolescent (mean age < 18 years) or adult (mean age ≥ 18 years), as developmental stage may influence responses to upward targets. Cultural background was coded based on the country in which the data were collected, categorized as either Western or Eastern, to examine potential cultural differences in reacting to upward comparison. Data collection year was coded as a continuous variable to preserve information and maximize statistical power for detecting potential temporal trends.

Because the included literature spanned cross-sectional, longitudinal, intensive longitudinal, and experimental samples, study method was additionally coded for exploratory analyses. To maintain interpretable subgroup sizes, designs were grouped into three broad categories: cross-sectional, longitudinal/intensive longitudinal, and experimental/quasi-experimental. These comparisons were treated as exploratory because the design groups were unbalanced and the non-cross-sectional categories remained relatively small, but they were included to directly address whether the overall effect appeared stronger in longitudinal than in cross-sectional designs.

### 2.6 Meta-analytic procedures

All statistical analyses were performed using the meta for package (version 4.8-0) in R. Pearson correlation coefficients were converted to Fisher's *z* scores prior to analysis to ensure variance stability, and results were transformed back to correlations for reporting. For outcomes where higher scores indicated positive psychological functioning—specifically, wellbeing and self-esteem—effect sizes were reverse-coded in the omnibus model so that positive values consistently represented a maladaptive impact of upward comparison. In single-outcome analyses, effect sizes are reported in their original direction to preserve interpretability against the source studies.

To appropriately account for the statistical dependence arising from multiple effect sizes extracted from the same study, a three-level random-effects meta-analytic model was employed (Cheung, 2014; Assink, 2016). This model partitions the total variance into three components: sampling variance at the level of the individual effect size (Level 1), within-study variance reflecting heterogeneity among effect sizes drawn from the same study (Level 2), and between-study variance reflecting heterogeneity across independent studies (Level 3). Compared with traditional approaches that aggregate dependent effect sizes into a single composite, the three-level model retains all eligible effect sizes from each study, thereby preserving statistical power while properly modeling the non-independence of estimates within studies. All models were fitted using the rma.mv() function with restricted maximum likelihood (REML) estimation. The necessity of the three-level structure was formally tested by comparing the three-level model against a reduced two-level model (omitting the within-study variance component) using a likelihood ratio test.

Importantly, because a random-effects meta-analysis estimates the mean of a distribution of true effects rather than a single underlying effect, we report the average estimated correlation as $\bar{r}$ throughout the manuscript. This notation reflects the inferential framework of random-effects models, in which heterogeneity among true effects is assumed and the model estimates an average across that distribution rather than a single fixed value.

Moderator analyses were conducted by fitting meta-regression models within the same three-level framework, adding moderators as fixed-effect predictors. Outcome type, age group, and cultural background were entered as categorical moderators, while data collection year was entered as a continuous predictor. In addition, exploratory design-based comparisons examined study method in the overall model and directly contrasted cross-sectional with longitudinal/intensive longitudinal samples. The omnibus test of moderators (*QM* statistic) was used to evaluate the overall significance of each moderator. Publication bias was evaluated using funnel plot inspection and Egger's regression test, both implemented within the three-level framework by including the standard error of each effect size as a moderator. We note that Egger's test detects only certain forms of small-study effects and asymmetry; the absence of a detected effect should not be interpreted as evidence that no publication bias exists.

### 2.7 Study quality assessment

We evaluated the methodological quality of the included samples using the Joanna Briggs Institute Critical Appraisal Checklist for Analytical Cross-Sectional Studies (Moola et al., 2020) as a common appraisal framework across the dataset. Given that the literature included cross-sectional, longitudinal, and experimental samples, the checklist was used to code comparable domains—sample selection, control of confounding factors, measurement of exposure, measurement of outcomes, and handling of missing data—on a low, high, or unclear basis according to the information reported in each study. Two authors independently evaluated each study and classified each domain as low risk, high risk, or unclear risk. Studies employing random sampling were coded as low risk for selection bias, whereas convenience samples were classified as unclear or high risk depending on the detailed reporting of participant characteristics. Regarding confounding factors, studies that statistically controlled for key demographic variables such as age and gender were considered low risk. Discrepancies in coding were resolved through discussion to ensure consistency.

## 3 Results

### 3.1 Overview of studies

The final meta-analysis comprised 94 effect sizes drawn from 54 independent samples, with a total sample size of 36,583 participants. The dataset represented a diverse range of cultural backgrounds, comprising samples from both Western and Eastern countries, and covered developmental stages ranging from adolescence to adulthood. Detailed PICOS-structured characteristics of all included studies are presented in Supplementary Table 1, and a full reference list of included studies is provided in the Supplementary material.

### 3.2 Publication bias

**Figure 2:**
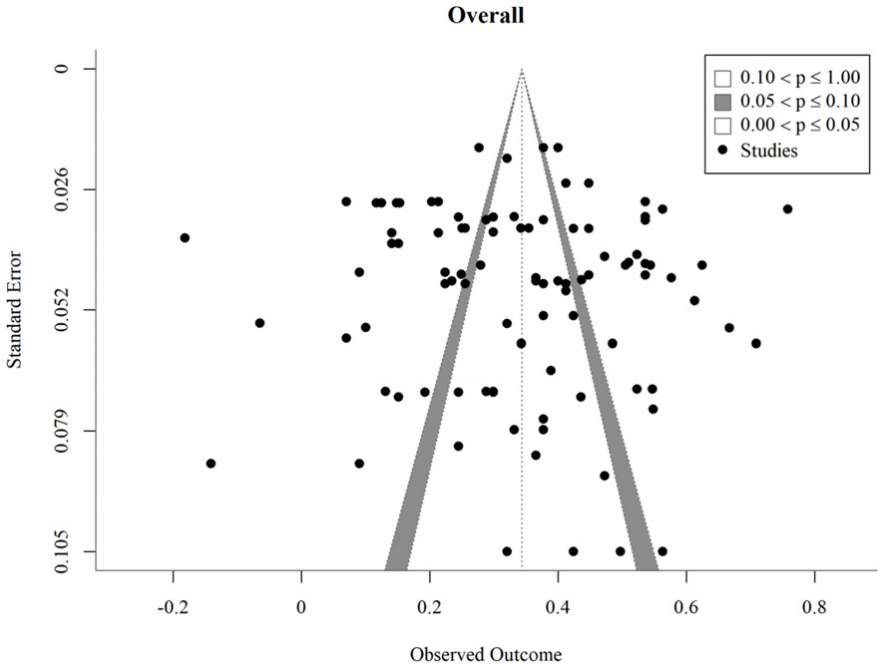
Funnel plot of standard errors by Fisher's z for the overall effect of upward social comparison on psychological outcomes.

Publication bias was assessed using Egger's regression test fitted within the three-level meta-analytic framework, with the standard error of each effect size entered as a moderator. For the overall dataset, Egger's test indicated no detected evidence of small-study effects (*z* = 0.23, *p* = 0.822). Consistent results were obtained for each outcome category: anxiety (*z* = −0.13, *p* = 0.899), depression (*z* = 0.70, *p* = 0.486), wellbeing (*z* = 1.47, *p* = 0.142), self-esteem (*z* = −0.71, *p* = 0.476), and social-evaluative negative emotions (*z* = 0.20, *p* = 0.840). The funnel plot for the overall analysis is shown in Figure 2, and funnel plots for each outcome category are presented in the Supplementary material. We emphasize that the absence of a detected small-study effect does not equate to the absence of publication bias. Egger's test has limited statistical power and only detects specific forms of asymmetry; the present results indicate no detected evidence of asymmetry rather than confirmed absence of bias. Furthermore, only published studies were included in the analyses, which represents an inherent constraint on the population of effect sizes synthesized here.

### 3.3 Quality assessment

**Figure 3:**
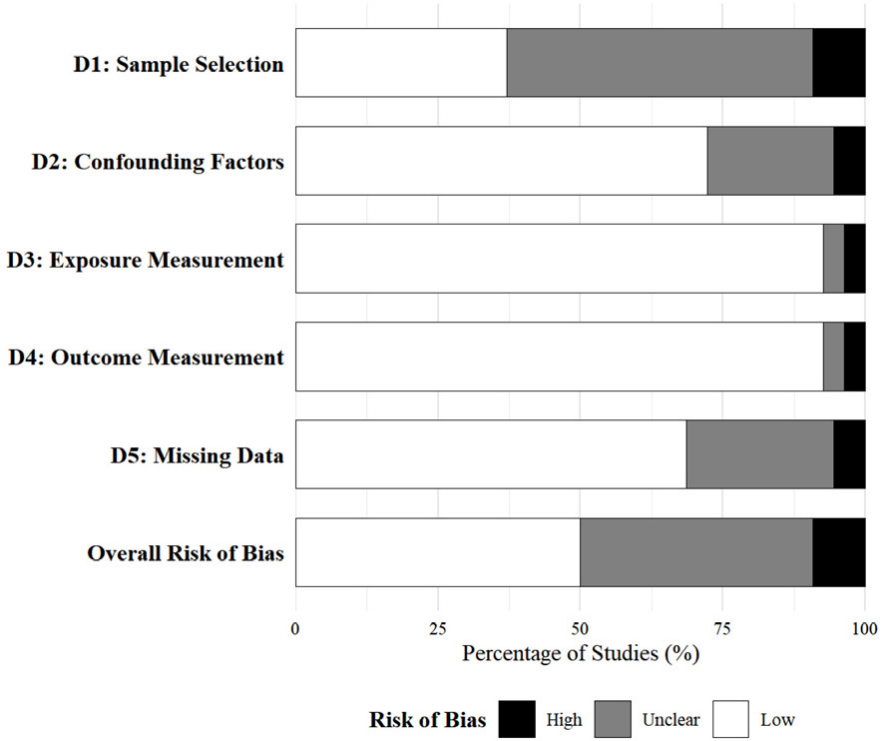
Risk of bias summary for the 54 included independent samples.

Figure 3 presents the summary of the risk of bias assessment across the 54 included independent samples. Overall, the methodological quality of the included studies was moderate. Regarding measurement validity, the majority of studies demonstrated a low risk of bias, given that the vast majority utilized validated psychometric scales to assess social media upward comparison and mental health outcomes. However, sample selection emerged as the primary source of potential bias, with confounding factor control and missing data handling being the secondary ones. For sample selection (D1), a considerable proportion of studies (53.7%) were rated as unclear risk of bias and 9.3% as high risk, which directly reflects the field's heavy reliance on convenience sampling strategies—most samples were recruited from university student populations or online user groups, leading to insufficient sample representativeness. For confounding factor control (D2), 72.2% of studies were classified as low risk for statistically controlling key demographic covariates such as age and gender, while the remaining 27.8% were rated as unclear (22.2%) or high (5.6%) risk due to the lack of rigorous covariate adjustment. For missing data handling (D5), 25.9% and 5.6% of studies were judged as unclear and high risk respectively, owing to the inadequate reporting or improper processing of missing data. In contrast, exposure measurement (D3) and outcome measurement (D4) showed an excellent methodological quality, with 92.6% of studies being low risk and only a small fraction (3.7% each) rated as unclear or high risk. Despite the above biases in sample selection and partial methodological domains, the predominance of low-risk ratings in the measurement domains supports the interpretability of the pooled estimates. Leave-one-out sensitivity analyses, conducted separately within each outcome domain, indicated that the average estimates remained stable when any single study was sequentially excluded (see Supplementary material for detailed results).

### 3.4 Overall analysis

A three-level random-effects model was fitted to the full dataset of 94 effect sizes nested within 54 independent samples (with self-esteem and wellbeing effect sizes reverse-coded for the omnibus analysis). The estimated average correlation between upward social comparison on social media and psychological maladjustment was significant ($\bar{r}$ =0.330, 95% CI [0.289, 0.370], *p* < 0.001). Variance decomposition indicated that 66.18% of the total random-effects variance was attributable to within-study heterogeneity (Level 2) and 33.82% to between-study heterogeneity (Level 3), with the remainder attributable to sampling error. A likelihood ratio test confirmed that the three-level model provided significantly better fit than a two-level model that omitted the within-study variance component (*p* < 0.001), supporting the appropriateness of the nested structure. As is appropriate for random-effects meta-analyses, this estimate represents the average of a distribution of true effects across studies rather than a single underlying effect, and individual study effects are expected to vary around this average.

To characterize average estimates by outcome domain, we first fit an outcome-type model parameterized without an intercept to obtain category-specific means. After reverse-coding positive outcomes for comparability, the average estimates were as follows: social-evaluative negative emotions, $\bar{r}$ = 0.438, 95% CI [0.385, 0.487]; anxiety, $\bar{r}$ = 0.382, 95% CI [0.322, 0.439]; depression, $\bar{r}$ = 0.306, 95% CI [0.252, 0.358]; wellbeing, $\bar{r}$ = 0.268, 95% CI [0.208, 0.326]; and self-esteem, $\bar{r}$ = 0.263, 95% CI [0.210, 0.314]. We then fit a reference-coded model using social-evaluative negative emotions as the reference category to formally test between-category differences. This omnibus test indicated significant overall differences across outcome categories (*QM*(4) = 47.84, *p* < 0.001). Relative to social-evaluative negative emotions, the average estimate for anxiety was not significantly different (*QM*(1) = 2.35, *p* = 0.126), whereas the average estimates for depression (*QM*(1) = 18.43, *p* < 0.001), wellbeing (*QM*(1) = 27.84, *p* < 0.001), and self-esteem (*QM*(1) = 31.02, *p* < 0.001) were significantly smaller. The category-specific estimates reported here were derived from the no-intercept means model and therefore differ slightly from the estimates obtained in the separate outcome-specific models reported in the sections below, which were fitted within each outcome subset and use the original (non-reverse-coded) effect-size direction.

Exploratory comparisons by study method did not indicate systematic differences in the overall reverse-coded association, *QM*(2) = 1.63, *p* = 0.443. The average estimates were similar for cross-sectional samples ($\bar{r}$ = 0.324, 95% CI [0.277, 0.370]) and longitudinal/intensive longitudinal samples ($\bar{r}$ = 0.322, 95% CI [0.221, 0.416]), whereas experimental/quasi-experimental samples showed a numerically larger but less precise estimate ($\bar{r}$ = 0.437, 95% CI [0.264, 0.582]). A direct comparison between cross-sectional and longitudinal/intensive longitudinal samples likewise was not significant, *QM*(1) = 0.002, *p* = 0.961, indicating that the available data did not support the hypothesis that the overall effect is stronger in longitudinal designs.

### 3.5 Anxiety

**Figure 4:**
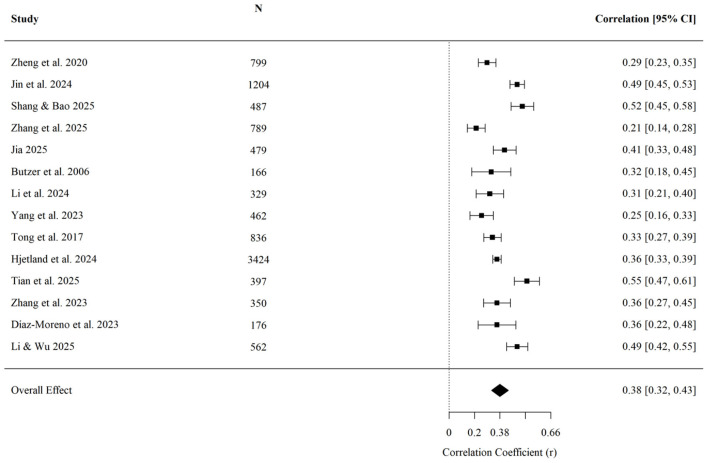
Forest plot of the effect of upward social comparison on anxiety.

On average, higher levels of upward comparison were associated with higher anxiety symptoms ($\bar{r}$ = 0.380, 95% CI [0.322, 0.435], *p* < 0.001; *k* = 14; Figure 4). Egger's regression test indicated no detected evidence of small-study effects (*z* = −0.13, *p* = 0.899). None of the examined moderators reached statistical significance: age (*QM* = 1.04, *p* = 0.307), cultural background (*QM* = 0.27, *p* = 0.601), or data collection year (*QM* = 1.51, *p* = 0.218). These results indicate that the available data did not detect moderation for anxiety, although the tests should be interpreted cautiously given the small subgroup sizes in some analyses.

### 3.6 Depression

**Figure 5:**
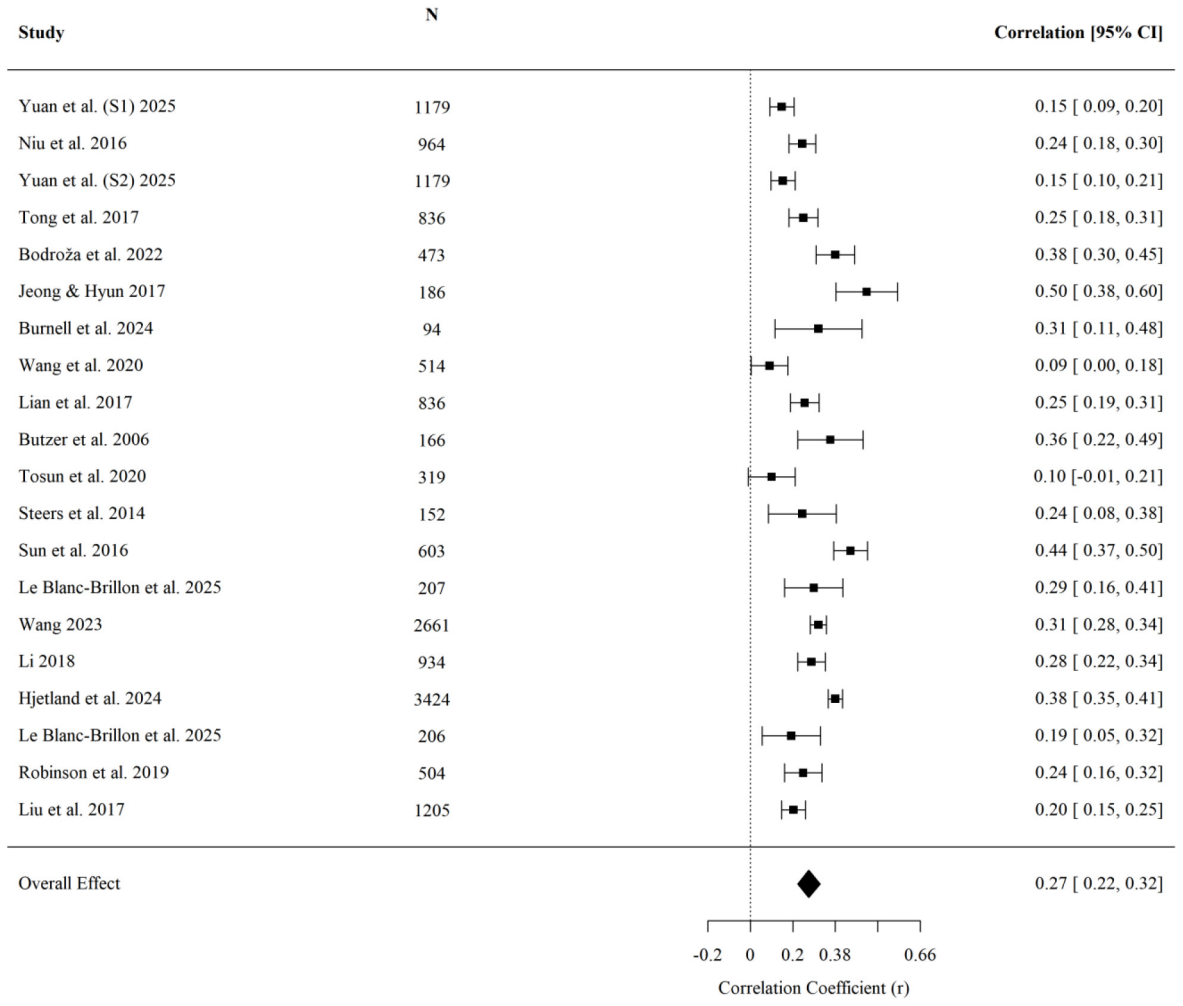
Forest plot of the effect of upward social comparison on depression.

For depression, upward comparison was on average positively associated with depressive symptoms ($\bar{r}$ = 0.268, 95% CI [0.219, 0.317], *p* < 0.001; *k* = 20; Figure 5). A sensitivity analysis excluding the study in which depression was operationalized as a binary major depressive disorder diagnosis and converted from an odds ratio yielded a nearly identical pooled estimate ($\bar{r}$ = 0.270, 95% CI [0.217, 0.321]), compared with the full-sample estimate ($\bar{r}$ = 0.268, 95% CI [0.219, 0.317]), indicating that the depression result was not driven by this study. Egger's regression test indicated no detected evidence of small-study effects (*z* = 0.70, *p* = 0.486). No significant moderating effects were detected for age (*QM* = 0.02, *p* = 0.899), cultural background (*QM* = 0.27, *p* = 0.601), or data collection year (*QM* = 1.91, *p* = 0.167). The available data therefore did not indicate moderation for depression across these coded contexts.

### 3.7 WellBeing

**Figure 6:**
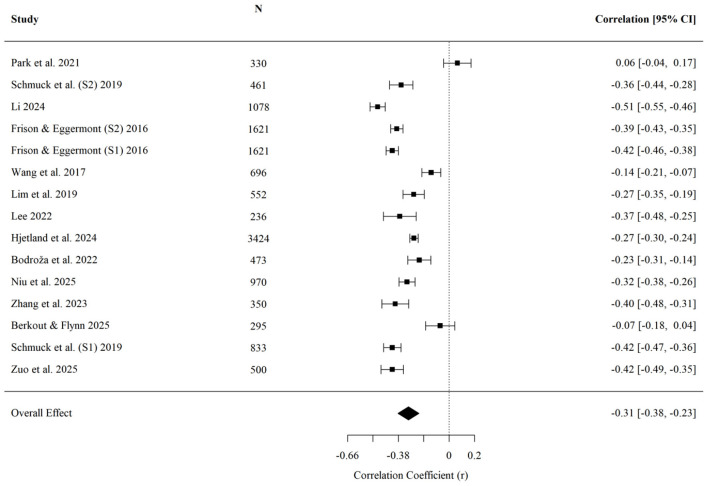
Forest plot of the effect of upward social comparison on wellbeing.

For wellbeing, higher levels of upward comparison were on average associated with lower levels of wellbeing ($\bar{r}$ = −0.310, 95% CI [−0.383, −0.234], *p* < 0.001; *k* = 15; Figure 6). Egger's regression test indicated no detected evidence of small-study effects (*z* = 1.47, *p* = 0.142). No significant moderating effects were detected for age (*QM* = 0.98, *p* = 0.323), cultural background (*QM* = 0.02, *p* = 0.893), or data collection year (*QM* = 0.00, *p* = 0.989).

### 3.8 Self-esteem

**Figure 7:**
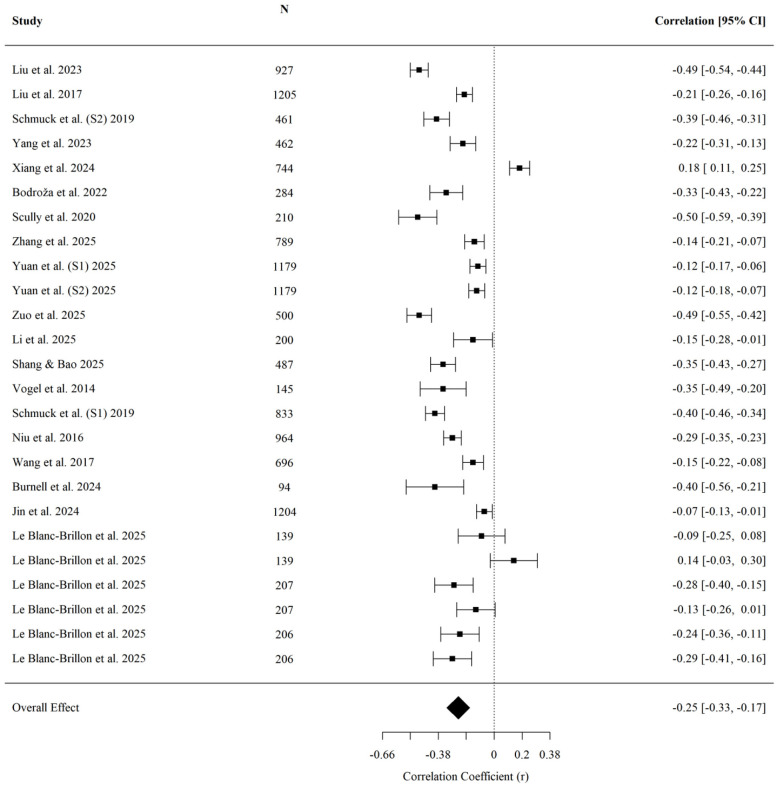
Forest plot of the effect of upward social comparison on self-esteem.

For self-esteem, higher levels of upward comparison were on average associated with lower self-esteem ($\bar{r}$ = −0.251, 95% CI [−0.326, −0.173], *p* < 0.001; *k* = 25; Figure 7). Egger's regression test indicated no detected evidence of small-study effects (*z* = −0.71, *p* = 0.476). No significant moderating effects were detected for age (*QM* = 0.00, *p* = 0.987), cultural background (*QM* = 2.15, *p* = 0.143), or data collection year (*QM* = 1.26, *p* = 0.262). Thus, the available data did not provide evidence of moderation for this outcome domain.

### 3.9 Social-evaluative negative emotions

**Figure 8:**
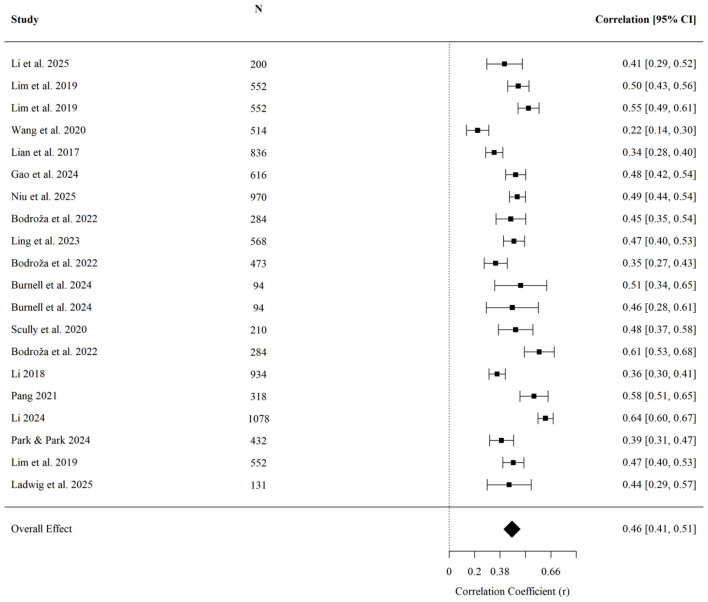
Forest plot of the effect of upward social comparison on social-evaluative negative emotions.

For social-evaluative negative emotions, upward comparison showed the largest average association in absolute magnitude among the five outcome categories examined ($\bar{r}$ = 0.458, 95% CI [0.406, 0.507], *p* < 0.001; *k* = 20; Figure 8). Egger's regression test indicated no detected evidence of small-study effects (*z* = 0.20, *p* = 0.840). None of the examined moderators reached statistical significance: age (*QM* = 0.85, *p* = 0.358), cultural background (*QM* = 0.03, *p* = 0.860), or data collection year (*QM* = 1.45, *p* = 0.229). Within the time window covered by the included studies, the available data did not indicate detectable moderation, including by data collection year.

## 4 Discussion

In this meta-analysis, we synthesized data from 54 independent samples involving 36,583 participants to estimate the average magnitude of associations between upward social comparison on social media and a range of psychological maladjustment indicators. By integrating the available evidence within a three-level random-effects framework, this study sought to characterize the average correlations observed across the literature, examine whether they vary with cultural background, age, and data collection year, describe how average estimates compare across outcome categories, and, in exploratory analyses, test whether the overall effect appeared stronger in longitudinal than in cross-sectional designs. To accomplish these aims, we tested three hypotheses. Hypothesis 1 predicted that upward social comparison would, on average, be associated with poorer psychological functioning across multiple mental health indicators. Hypothesis 2 examined whether the magnitude of these associations varies as a function of cultural background, age group, and data collection year. Hypothesis 3 examined whether average effect sizes differ across outcome categories, with social-evaluative negative emotions expected to show numerically larger estimates than more generalized outcome domains.

Consistent with Hypothesis 1, the omnibus three-level model yielded a significant positive average association between upward social comparison and psychological maladjustment after reverse-coding the positively-valenced outcomes. On average, higher levels of upward comparison were associated with higher levels of social-evaluative negative emotions, anxiety, and depression, and with lower levels of self-esteem and wellbeing (Vogel et al., 2014; Liu et al., 2017; Wang et al., 2020; Lee, 2022; Li, 2024; Zuo and Zan, 2025). These average associations are theoretically consistent with the Contrast Effect (Festinger, 1954), according to which the perceived discrepancy between one's actual self and the idealized self-presentation of others may foster a sense of relative deprivation (Steers et al., 2014; Li, 2024). Unlike offline interactions where context is often visible, digital comparison typically lacks mitigating cues, which may contribute to the average pattern observed here. The three-level analysis suggests that the average association extends across multiple domains of mental health rather than being confined to a single outcome (McComb et al., 2023; Berkout and Flynn, 2025). We caution, however, that the random-effects framework estimates an average across heterogeneous true effects, and the magnitude of any individual effect is expected to vary substantially around this average.

These findings also help clarify a broader debate in the social media and wellbeing literature. Meta-analyses based on global indicators such as overall social media use or time spent online often yield weak, mixed, or near-zero average associations with wellbeing, in part because such indicators collapse across qualitatively different forms of engagement (Liu and Baumeister, 2016; Saiphoo and Vahedi, 2019; Yoon et al., 2019; Hancock et al., 2022). By contrast, the present meta-analysis focuses on one psychologically specific process—upward social comparison—and shows that this process is reliably associated with poorer functioning across multiple domains (Appel et al., 2016; Verduyn et al., 2020; McComb et al., 2023). This distinction may help reconcile broader disagreements about whether social media is uniformly harmful: average effects based on global use indicators and effects tied to specific engagement processes should not be treated as interchangeable. Related intervention work also suggests that changing how individuals engage with social media may be a more actionable target than focusing on exposure in the abstract (Andrade et al., 2023). Our contribution is therefore not to argue that all social media use is uniformly harmful, but to identify one mechanism through which particular forms of engagement may undermine wellbeing and may offer a more actionable target for intervention.

The residual heterogeneity observed in the present models is also likely to reflect variability in study design and measurement. The included literature spans cross-sectional, longitudinal, intensive longitudinal, and experimental approaches, and measures of upward comparison ranged from established psychometric scales to study-specific items, not all of which appear to have been fully validated. Such variability may account for part of the unexplained variance that was not captured by the moderators tested here and should be considered when interpreting the pooled estimates.

Contrary to Hypothesis 2, none of the examined moderators reached statistical significance in the outcome-specific models. Across anxiety, depression, wellbeing, self-esteem, and social-evaluative negative emotions, the available data did not provide evidence that the average magnitude of the associations varied systematically as a function of cultural background, age group, or data collection year. Exploratory design-based comparisons likewise did not indicate stronger overall effects in longitudinal than in cross-sectional studies (*QM*(1) = 0.002, *p* = 0.961), nor did the broader three-category study-method comparison detect significant between-method differences (*QM*(2) = 1.63, *p* = 0.443). These results should be interpreted cautiously: tests of moderation can have limited statistical power, subgroup distributions were uneven in several outcome domains, and the available cultural, developmental, and design-based codings remained relatively coarse. Overall, the findings suggest that the association between upward comparison and psychological maladjustment is observable across the included contexts, while leaving open the possibility that more fine-grained contextual differences may emerge in future research.

Contrary to our expectation that the magnitude of these associations might have intensified in recent years following the spread of algorithm-driven short-video platforms, data collection year did not emerge as a significant moderator for any outcome category when modeled as a continuous predictor in the three-level framework. This result does not support the hypothesis of a recent escalation, but it should not be taken as conclusive evidence that the digital comparison environment has remained unchanged. The available studies span a relatively narrow temporal window, and meta-regression with year as a continuous predictor may have limited power to detect gradual shifts. Future research with longer time spans and finer temporal coding will be needed to clarify whether and how the psychological correlates of upward comparison have evolved alongside structural changes in social media platforms.

Similarly, no significant moderating effect of age was detected for any outcome category. The available data do not indicate that adolescents and young adults differ in the average magnitude of their associations between upward comparison and the examined outcomes. While adolescents face developmental tasks such as identity formation and peer acceptance, young adults face parallel pressures related to career milestones, financial status, and relationship success (Jin et al., 2024). The present results are consistent with the view that the relevant comparison processes operate across these developmental stages, although the binary age coding used here is coarse and more granular developmental coding may yield different results in future work.

Partially consistent with Hypothesis 3, formal between-category comparisons indicated overall differences across outcome domains (*QM*(4) = 47.84, *p* < 0.001). In the cross-domain comparison model, positively valenced outcomes were reverse-coded for comparability; under this parameterization, the largest average estimate was observed for social-evaluative negative emotions ($\bar{r}$ = 0.438), followed by anxiety ($\bar{r}$ = 0.382), depression ($\bar{r}$ = 0.306), lower wellbeing ($\bar{r}$ = 0.268), and lower self-esteem ($\bar{r}$ = 0.263). When social-evaluative negative emotions were used as the reference category, their average estimate did not differ significantly from anxiety (*QM*(1) = 2.35, *p* = 0.126), but it was significantly larger than the average estimates for depression (*QM*(1) = 18.43, *p* < 0.001), lower wellbeing (*QM*(1) = 27.84, *p* < 0.001), and lower self-esteem (*QM*(1) = 31.02, *p* < 0.001). This pattern is descriptively consistent with the proximal-distal organizing framework outlined in the Introduction, which groups envy, body dissatisfaction, fear of negative evaluation, and loneliness together as social-evaluative reactions theoretically more proximal to comparison processes. We emphasize, however, that the present analyses test moderation, not mediation or temporal precedence; the difference in average estimates does not constitute a confirmatory test of a strict proximal-distal hierarchy. The comparatively larger average estimate for social-evaluative negative emotions is also consistent with theoretical accounts according to which acute affective responses to specific comparison content—such as body dissatisfaction following exposure to idealized physiques—may be more proximally tied to the comparison episode than diffuse symptoms such as generalized anxiety or depression (Xiang and Kong, 2024; Li, 2024).

These findings also speak to the role of social media platform design in shaping the psychological consequences of upward comparison. The relatively larger average association observed for social-evaluative negative emotions may reflect specific design features of contemporary social media platforms. Algorithmically curated feeds, image-centric interfaces, and engagement-based ranking systems collectively expose users to a high density of idealized self-presentations, which may facilitate frequent and salient upward comparisons (Verduyn et al., 2020). At the same time, design features such as quantifiable social metrics—likes, follower counts, and similar indicators—translate ambiguous social standing into explicit numerical indicators, which may intensify the affective consequences of perceived inferiority. Visual-centric platforms such as Instagram and TikTok may particularly invite habitual social monitoring (Bodroza et al., 2022; Hjetland et al., 2024). We note, however, that the present meta-analysis cannot directly test the contribution of specific design features, and these interpretations should be regarded as theoretically motivated rather than empirically established. Future research that systematically varies platform features, or that compares users across different platform ecosystems, would provide more direct evidence on this question.

## 5 Limitations and future directions

The interpretation of our findings should be considered in light of several methodological limitations. Most prominently, the included studies are predominantly cross-sectional, which precludes causal inferences (Zhang et al., 2023; Shang and Bao, 2025). While our theoretical framing is consistent with the possibility that upward comparison contributes to psychological maladjustment, it is also plausible that a reciprocal pattern exists, in which individuals with higher trait envy or depression may engage in more upward comparison (Verduyn et al., 2020). Although recent experimental work provides some causal evidence, future research would benefit from longitudinal cross-lagged panel designs that can disentangle the temporal ordering of these effects (Jia, 2025; Yuan et al., 2025).

Relatedly, although we conducted exploratory design-based comparisons, these analyses were constrained by the imbalance of the design groups. The vast majority of effect sizes came from cross-sectional studies, whereas longitudinal/intensive longitudinal and experimental/quasi-experimental samples were comparatively sparse. Accordingly, the null design comparisons should not be interpreted as definitive evidence that study method is irrelevant; larger and more balanced sets of longitudinal and experimental studies will be needed to test this question more rigorously and to determine whether design-sensitive differences emerge under better-powered conditions.

A related concern pertains to the simplification of our cultural classification, in which cultural background was operationalized as a binary Western vs. Eastern dichotomy. Although cultural background did not emerge as a significant moderator in the present analyses, this broad dichotomy may still mask more specific cultural values—such as vertical vs. horizontal individualism—that could shape how upward comparison is appraised (Vogel et al., 2014; Liu et al., 2017). The absence of detected moderation under this coarse coding should therefore not be interpreted as evidence that culture is irrelevant. Future research would benefit from finer-grained cultural coding and from explicit examination of protective factors such as self-concept clarity and growth mindset across different cultural frameworks (Li, 2024; Xiang and Kong, 2024).

In addition, although the search string was designed to cover a broad range of psychological outcome domains, the selection of any finite set of keywords inevitably constrains the scope of retrieved literature. Studies using measurement instruments or conceptual labels that fall outside the chosen terms may not have been captured. Future meta-analyses with even more comprehensive keyword coverage, particularly for outcomes beyond the internalizing symptom spectrum, would provide stronger evidence across a wider range of psychological outcomes.

Furthermore, only published studies were included in the meta-analysis. While Egger's regression tests within the three-level framework did not detect evidence of small-study effects in any outcome category, we emphasize that such tests have limited statistical power and that the absence of detected effects does not equate to the absence of publication bias. The estimates presented here therefore characterize the published literature, which may differ systematically from the full population of conducted studies. Relatedly, we did not contact the authors of studies that did not report a usable effect size; some of these studies may have contained additional information from which a correlation coefficient could have been computed, and their omission represents a further constraint on the comprehensiveness of the literature synthesized here.

Finally, we note that the random-effects framework estimates the mean of a distribution of heterogeneous true effects rather than a single underlying effect. Variance decomposition in our three-level model indicated substantial between-study and within-study heterogeneity, which is consistent with the view that the magnitude of these associations varies meaningfully across studies and contexts. Readers should therefore interpret the average estimates reported here as descriptive summaries of a distribution of effects, not as point estimates of a single fixed parameter.

## 6 Conclusion

This meta-analysis synthesized current empirical evidence to estimate the average magnitude of associations between upward social comparison on social media and a range of psychological maladjustment indicators using a three-level random-effects framework. On average, upward comparison was associated with higher social-evaluative negative emotions, anxiety, and depression, and with lower wellbeing and self-esteem. In the cross-domain comparison model, the largest average estimate was observed for social-evaluative negative emotions. Among the primary moderators examined, no significant moderation by age, cultural background, or data collection year was detected for any outcome category. Exploratory design-based comparisons likewise did not indicate stronger overall effects in longitudinal than in cross-sectional studies. These results indicate that upward comparison on social media is, on average, associated with multiple indicators of psychological maladjustment, while also underscoring the substantial heterogeneity in the magnitude of these associations across studies and the limitations of the current evidence base for drawing causal or universal conclusions. We hope these findings will inform both future research on the contextual moderators of digital comparison and the design of culturally tailored interventions to support psychological wellbeing in an environment increasingly shaped by curated online self-presentation.

## Data Availability

The datasets analyzed for this study are available in the OSF (Open Science Framework) repository at: https://osf.io/ah9yj?view_only=79ddf74a714c4158b4213c4d155e57f4.
