# Supplementary material for: “Looking up” linked to feeling down: a meta-analysis of online upward social comparison and psychological maladjustment
Source: Front Psychol. 2026 May 15;17:1825169. doi: 10.3389/fpsyg.2026.1825169 (PMC13219356; doi:10.3389/fpsyg.2026.1825169)
Supplement: Supplementary file 1 [file Data_Sheet_1.zip › Supplementary Material/Reference_list_of_included_studies.docx]

**Reference list of included studies**

Berkout, O.V., and Flynn, M.K. (2025). Social media utilization, fear of missing out, upward social comparison, and self-compassion as predictors of psychological distress and life satisfaction among individuals with mental health concerns. *Journal of Technology in Behavioral Science* 10(1)**,** 7–14. <https://doi.org/10.1007/s41347-024-00411-z>.

Bodroza, B., Obradovic, V., and Ivanovic, S. (2022). Active and passive selfie-related behaviors: Implications for body image, self-esteem and mental health. *Cyberpsychology: Journal of Psychosocial Research on Cyberspace* 16(2), Article 3. https://doi.org/10.5817/CP2022-2-3.

Burnell, K., Trekels, J., Prinstein, M.J., and Telzer, E.H. (2024). Adolescents’ social comparison on social media: Links with momentary self-evaluations. *Affective Science* 5(4)**,** 295–299. https://doi.org/10.1007/s42761-024-00240-6.

Butzer, B., and Kuiper, N.A. (2006). Relationships between the frequency of social comparisons and self-concept clarity, intolerance of uncertainty, anxiety, and depression. *Personality and Individual Differences* 41(1)**,** 167–176. <https://doi.org/10.1016/j.paid.2005.12.017>.

Diaz-Moreno, A., Bonilla, I., and Chamarro, A. (2023). Negative social comparison: The influence of anxiety, emotion regulation and problematic social media use. *Ansiedad y Estrés – Anxiety and Stress* 29(3)**,** 181–186. https://doi.org/10.5093/anyes2023a221134-7937.

Frison, E., and Eggermont, S. (2016). “Harder, better, faster, stronger”: Negative comparison on Facebook and adolescents’ life satisfaction are reciprocally related. *Cyberpsychology, Behavior, and Social Networking* 19(3)**,** 158–164. <https://doi.org/10.1089/cyber.2015.0296>.

Gao, B., Shen, Q., Lu, J., Xu, Y., and Wu, J. (2024). Why can’t I stop buying? Upward social comparison on social networking sites and online compulsive buying: A latent moderated mediation model. *Current Psychology* 43(8)**,** 7059–7070. https://doi.org/10.1007/s12144-023-04891-9.

Hjetland, G., Finseras, T., Sivertsen, B., Colman, I., Hella, R., Andersen, A., et al. (2024). Digital self-presentation and adolescent mental health: Cross-sectional and longitudinal insights from the “LifeOnSoMe”-study. *BMC Public Health* 24, 2635. https://doi.org/10.1186/s12889-024-20052-4.

Jeong, S. R., and Hyun, M. H. (2017). The mediating effect of upward social comparison moderated by control flexibility on the relationship between social network service use intensity and depression: Focused on Instagram. *The Korean Journal of Health Psychology* 22(4)**,** 1035–1053. <https://doi.org/10.17315/kjhp.2017.22.4.011>.

Jia, Z. (2025). An experimental study on the impact of social media images on users’ online social anxiety in China. *Discover Psychology* 5(1)**,** 14. https://doi.org/10.1007/s44202-025-00337-4.

Jin, T., Chen, Y., and Zhang, K. (2024). Effects of social media use on employment anxiety among Chinese youth: The roles of upward social comparison, online social support and self-esteem. *Frontiers in Psychology* 15, 1398801. https://doi.org/10.3389/fpsyg.2024.1398801.

Ladwig, G., Schönhals, K., Tanck, J., Quittkat, H., and Vocks, S. (2025). Upward comparison predicts an increase in state body dissatisfaction after fitspiration exposure. *Scientific Reports* 15, 42936. https://doi.org/10.1038/s41598-025-29830-5.

Le Blanc-Brillon J, Fortin J-S, Lafrance L and Hétu S. (2025). The associations between social comparison on social media and young adults’ mental health. *Frontiers in Psychology* 16, 1597241. https://doi.org/10.3389/fpsyg.2025.1597241

Lee, J.K. (2022). The effects of social comparison orientation on psychological well-being in social networking sites: Serial mediation of perceived social support and self-esteem. *Current Psychology* 41(9)**,** 6247–6259. <https://doi.org/10.1007/s12144-020-01114-3>.

Li, J., and Wu, J. (2025). Understanding young adults’ social media anxiety: Mediating role of upward social comparison and the moderating role of psychological resilience. *International Journal of Mental Health Promotion* 27(12)**,** 1883–1896. https://doi.org/10.32604/ijmhp.2025.071306.

Li, M., Li, X., Yang, F., and Zhang, T. (2025). They are more beautiful than me! How social media use increases women’s body-related envy and cosmetic surgery consideration. *Frontiers in Psychology* 16, 1628208. https://doi.org/10.3389/fpsyg.2025.1628208.

Li, S., Zhang, K., Kong, K., Niu, H., Jiang, Z., and Guo, H. (2024). Upward social comparison on network and alcohol dependence in college students: The chain mediation impacts of relative deprivation and anxiety. *BMC Psychology* 12(1)**,** 756. <https://doi.org/10.1186/s40359-024-02259-7>.

Li, Y. (2018). Upward social comparison and depression in social network settings: The roles of envy and self-efficacy. *Internet Research* 29(1)**,** 46–59. https://doi.org/10.1108/IntR-09-2017-0358.

Li, Y. (2024). Upward social comparisons on social networking sites (SNSs) and subjective well-being: The mediating role of envy. *Journal of Psychology in Africa* 34(5)**,** 508–513. https://doi.org/10.1080/14330237.2024.2425231.

Lian, S.-L., Sun, X.-J., Niu, G.-F., and Zhou, Z.-K. (2017). Upward social comparison on social network sites and depression: A moderated mediation model and gender difference. *Acta Psychologica Sinica* 49(7)**,** 941–952. <https://doi.org/10.3724/SP.J.1041.2017.00941>.

Lim, M., and Yang, Y. (2019). Upward social comparison and Facebook users’ grandiosity. *Online Information Review* 43(4)**,** 635–652. <https://doi.org/10.1108/OIR-04-2017-0137>.

Ling, Y., Gao, B., Jiang, B., Fu, C., and Zhang, J. (2023). Materialism and envy as mediators between upward social comparison on social network sites and online compulsive buying among college students. *Frontiers in Psychology* 14, 1085344. https://doi.org/10.3389/fpsyg.2023.1085344.

Liu, Q.-Q., Zhou, Z.-K., Yang, X.-J., Niu, G.-F., Tian, Y., and Fan, C.-Y. (2017). Upward social comparison on social network sites and depressive symptoms: A moderated mediation model of self-esteem and optimism. *Personality and Individual Differences* 113**,** 223–228. https://doi.org/10.1016/j.paid.2017.03.037.

Liu, Y., Chi, X., and Xin, X. (2023). Storing, not reading: Investigating the link between upward social comparison via social media and digital hoarding behavior in Chinese youth. *Psychology Research and Behavior Management* 16, 5209–5224. <https://doi.org/10.2147/PRBM.S441859>.

Niu, G.-F., Sun, X.-J., Zhou, Z.-K., Kong, F., and Tian, Y. (2016). The impact of social network site (Qzone) on adolescents’ depression: The serial mediation of upward social comparison and self-esteem. *Acta Psychologica Sinica* 48(10)**,** 1282–1291. <https://doi.org/10.3724/SP.J.1041.2016.01282>.

Niu, X., Gou, L.X., Han, Y., Zhou, X., and Wang, J.L. (2025). Self-concept clarity and envy as mediators between upward social comparison on social networking sites and subjective well-being. *British Journal of Developmental Psychology* 43(3)**,** 611–628. https://doi.org/10.1111/bjdp.12538.

Pang, H. (2021). Unraveling the influence of passive and active WeChat interactions on upward social comparison and negative psychological consequences among university students. *Telematics and Informatics* 57, 101510. https://doi.org/10.1016/j.tele.2020.101510

Park, H., and Park, Y. (2024). Negative upward comparison and relative deprivation: Sequential mediators between social networking service usage and loneliness. *Current Psychology* 43(10)**,** 9141–9151. https://doi.org/10.1007/s12144-023-05057-3.

Park, J., Kim, B., and Park, S. (2021). Understanding the behavioral consequences of upward social comparison on social networking sites: The mediating role of emotions. *Sustainability* 13(11)**,** 5781. https://doi.org/10.3390/su13115781.

Robinson, A., Bonnette, A., Howard, K., Ceballos, N., Dailey, S., Lu, Y. and Grimes, T. (2019). Social comparisons, social media addiction, and social interaction: An examination of specific social media behaviors related to major depressive disorder in a millennial population. *Journal of Applied Biobehavioral Research*, 24(1), e12158. https://doi.org/10.1111/jabr.12158

Schmuck, D., Karsay, K., Matthes, J., and Stevic, A. (2019). “Looking up and feeling down”: The influence of mobile social networking site use on upward social comparison, self-esteem, and well-being of adult smartphone users. *Telematics and Informatics* 42**,** 101240. https://doi.org/10.1016/j.tele.2019.101240.

Scully, M., Swords, L. and Nixon, E. (2023). Social comparisons on social media: online appearance-related activity and body dissatisfaction in adolescent girls. *Irish Journal of Psychological Medicine* 40(1), pp. 31–42. https://doi.org/10.1017/ipm.2020.93

Shang, A., and Bao, H. (2025). Self-esteem and appearance anxiety among Chinese college students: The roles of social media use and upward social comparison. *Frontiers in Psychology* 16**,** 1562711. https://doi.org/10.3389/fpsyg.2025.1562711.

Steers, M.-L.N., Wickham, R.E., and Acitelli, L.K. (2014). Seeing everyone else’s highlight reels: How Facebook usage is linked to depressive symptoms. *Journal of Social and Clinical Psychology* 33(8)**,** 701–731. <https://doi.org/10.1521/jscp.2014.33.8.701>.

Sun, X.-J., Lian, S.-L., Niu, G.-F., Yan, J.-L., Tong, Y.-T., and Zhou, Z.-K. (2016). Social network site use and depression in adolescents: Mediating of upward social comparison. *Chinese Journal of Clinical Psychology* 24(1)**,** 32–35. https://doi.org/10.16128/j.cnki.1005-3611.2016.01.007.

Tian, J., Li, B., and Zhang, R. (2025). The impact of upward social comparison on social media on appearance anxiety: A moderated mediation model. *Behavioral Sciences* 15(1)**,** 8. https://doi.org/10.3390/bs15010008.

Tong, Y.-T., Qiu, X.-W., Lian, S.-L., and Zhang, M.-M. (2017). Upward social comparison in social network site and depression: Mediating of social anxiety. *Chinese Journal of Clinical Psychology* 25(3)**,** 498–501. https://doi.org/10.16128/j.cnki.1005-3611.2017.03.022.

Tosun, L., and Kasdarma, E. (2020). Passive Facebook use and depression: A study of the roles of upward comparisons, emotions, and friendship type. *Journal of Media Psychology: Theories, Methods, and Applications* 32(4)**,** 165–175. https://doi.org/10.1027/1864-1105/a000269.

Vogel, E.A., Rose, J.P., Roberts, L.R., and Eckles, K. (2014). Social comparison, social media, and self-esteem. *Psychology of Popular Media Culture* 3(4)**,** 206–222. <https://doi.org/10.1037/ppm0000047>.

Wang, J., Wang, H., Gaskin, J., and Hawk, S. (2017). The mediating roles of upward social comparison and self-esteem and the moderating role of social comparison orientation in the association between social networking site usage and subjective well-being. *Frontiers in Psychology* 8**,** 771. https://doi.org/10.3389/fpsyg.2017.00771.

Wang, R., Cong, S., Sha, L., Sun, X., Zhu, R., Feng, J., et al. (2023). Association between social networking site use intensity and depression among Chinese pregnant women: Cross-sectional study. *Journal of Medical Internet Research* 25**,** e41793. <https://doi.org/10.2196/41793>.

Wang, W., Wang, M., Hu, Q., Wang, P., Lei, L., and Jiang, S. (2020). Upward social comparison on mobile social media and depression: The mediating role of envy and the moderating role of marital quality. *Journal of Affective Disorders* 270**,** 143–149. https://doi.org/10.1016/j.jad.2020.03.173.

Xiang, K., and Kong, F. (2024). Passive social networking sites use and disordered eating behaviors in adolescents: The roles of upward social comparison and body dissatisfaction and its sex differences. *Appetite* 198**,** 107360. https://doi.org/10.1016/j.appet.2024.107360.

Yang, F., Li, M., and Han, Y. (2023). Whether and how will using social media induce social anxiety? The correlational and causal evidence from Chinese society. *Frontiers in Psychology* 14**,** 1217415. https://doi.org/10.3389/fpsyg.2023.1217415.

Yuan, M., Sun, Y., Wang, Y., and Yu, L. (2025). The influence of upward social comparison on social network sites on middle school students’ depression: A two-wave multiple mediation model. *Development and Psychopathology* 37(3)**,** 1685–1693. https://doi.org/10.1017/s0954579424001913.

Zhang, P., Wang, M., Ding, L., Liu, J., Yuan, Y., Zhang, J., et al. (2025). The effect of self-esteem on mobile phone addiction among college students: Sequential mediating effects of online upward social comparison and social anxiety. *Psychology Research and Behavior Management* 18**,** 657–669. https://doi.org/10.2147/prbm.S508668.

Zhang, W., Jiang, F., Zhu, Y., and Zhang, Q. (2023). Risks of passive use of social network sites in youth athletes: A moderated mediation analysis. *Frontiers in Psychology* 14**,** 1219190. https://doi.org/10.3389/fpsyg.2023.1219190.

Zheng, Y., Yang, X., Zhou, R., Niu, G., Liu, Q., and Zhou, Z. (2020). Upward social comparison and state anxiety as mediators between passive social network site usage and online compulsive buying among women. *Addictive Behaviors* 111**,** 106569. https://doi.org/10.1016/j.addbeh.2020.106569.

Zuo, F., and Zan, Q. (2025). Cyber upward social comparison and well-being among college students: The chain mediating roles of self-esteem and emotional regulation. *BMC Psychology* 13**,** 1202. https://doi.org/10.1186/s40359-025-03521-2.
