# Supplementary material for: “Looking up” linked to feeling down: a meta-analysis of online upward social comparison and psychological maladjustment
Source: Front Psychol. 2026 May 15;17:1825169. doi: 10.3389/fpsyg.2026.1825169 (PMC13219356; doi:10.3389/fpsyg.2026.1825169)
Supplement: Supplementary file 1 [file Data_Sheet_1.zip › Supplementary Material/Supplementary_Table_1.docx]

**Supplementary Table 1. Characteristics of Studies Included in the Meta-Analysis**

| **ID** | **Author (Year)** | **Population (Age group)** | **Country / Cultural Background** | **Sample Size (N)** | **Data Collection Year** | **Exposure (I)** | **Comparator (C)** | **Outcomes (O)** | **Study Design (S)** | **k** |
| --- | --- | --- | --- | --- | --- | --- | --- | --- | --- | --- |
| 1 | Bodroža et al. (2022) | Adult | Western | 284 | 2018 | Upward social comparison on social media — SRBEQ (Upward Appearance Comparison subscale) | Within-subject variation (no between-group comparator) | Social-Evaluative Negative Emotions; Self-Esteem | Cross-sectional | 3 |
| 2 | Bodroža et al. (2022) | Adult | Western | 473 | 2018 | Upward social comparison on social media — SRBEQ (Upward Appearance Comparison subscale) | Within-subject variation (no between-group comparator) | Depression; Social-Evaluative Negative Emotions; Well-Being | Cross-sectional | 3 |
| 3 | Burnell et al. (2024) | Adolescent | Western | 94 | 2021 | Upward social comparison on social media — Single-item momentary comparison rating | Within-subject variation (no between-group comparator) | Depression; Social-Evaluative Negative Emotions; Self-Esteem | Ecological momentary assessment | 4 |
| 4 | Jia (2025) | Adult | Eastern | 479 | 2024 | Upward social comparison on social media — Gibbons & Buunk INCOM | Control / no-exposure condition | Anxiety | Experimental | 1 |
| 5 | Díaz-Moreno et al. (2023) | Adult | Spain (Western) | 176 | 2023 | Upward social comparison on social media — Negative Social Media Comparison Scale (NSMCS) | Within-subject variation (no between-group comparator) | Anxiety | Cross-sectional | 1 |
| 6 | Niu et al. (2025) | Adult | China (Eastern) | 970 | 2023 | Upward social comparison on social media — Lee (2014) Upward Social Comparison Scale | Within-subject variation (no between-group comparator) | Social-Evaluative Negative Emotions; Well-Being | Cross-sectional | 2 |
| 7 | Wang (2023) | Adult | Eastern | 2661 | 2021 | Upward social comparison on social media — Adapted Iowa–Netherlands Comparison Orientation Measure | Within-subject variation (no between-group comparator) | Depression | Cross-sectional | 1 |
| 8 | Zuo et al. (2025) | Adult | China (Eastern) | 500 | 2025 | Upward social comparison on social media — INCOM Chinese Upward Social Comparison version (INCOM-CUSC) | Within-subject variation (no between-group comparator) | Self-Esteem; Well-Being | Cross-sectional | 2 |
| 9 | Hjetland et al. (2024) | Adolescent | Norway (Western) | 3424 | 2020 | Upward social comparison on social media — Social media Photo Activity and Upward Social Comparison Inventory (SPAUSCIS) | Within-subject variation (no between-group comparator) | Anxiety; Depression; Well-Being | Cross-sectional + longitudinal | 3 |
| 10 | Jin et al. (2024) | Adult | China (Eastern) | 1204 | 2023 | Upward social comparison on social media — INCOM upward comparison subscale | Within-subject variation (no between-group comparator) | Anxiety; Self-Esteem | Cross-sectional | 2 |
| 11 | Butzer et al. (2006) | Adult | Canada (Western) | 166 | 2005 | Upward social comparison on social media — Revised INCOM (upward comparison) | Within-subject variation (no between-group comparator) | Anxiety; Depression | Cross-sectional | 2 |
| 12 | Zhang et al. (2023) | Adolescent | China (Eastern) | 350 | 2022 | Upward social comparison on social media — Revised De Vries & Kühne scale | Within-subject variation (no between-group comparator) | Anxiety; Well-Being | Cross-sectional | 2 |
| 13 | Shang & Bao (2025) | Adult | Eastern | 487 | 2024 | Upward social comparison on social media — Upward social comparison scale | Within-subject variation (no between-group comparator) | Anxiety; Self-Esteem | Cross-sectional | 2 |
| 14 | Vogel et al. (2014) | Adult | Western | 145 | 2013 | Upward social comparison on social media — Self-developed upward comparison questionnaire | Within-subject variation (no between-group comparator) | Self-Esteem | Cross-sectional | 1 |
| 15 | Scully et al. (2023) | Adolescent | Western | 210 | 2020 | Upward social comparison on social media — Adapted upward comparison measure (no validated scale specified) | Within-subject variation (no between-group comparator) | Social-Evaluative Negative Emotions; Self-Esteem | Cross-sectional | 2 |
| 16 | Berkout & Flynn (2025) | Adult | USA (Western) | 295 | 2024 | Upward social comparison on social media — Single-item upward social comparison measure | Within-subject variation (no between-group comparator) | Well-Being | Cross-sectional | 1 |
| 17 | Tosun et al. (2020) | Adult | Türkiye (Western) | 319 | 2018 | Upward social comparison on social media — Self-developed upward Facebook comparison scale | Within-subject variation (no between-group comparator) | Depression | Cross-sectional | 1 |
| 18 | Steers et al. (2014) | Adult | USA (Western) | 152 | 2013 | Upward social comparison on social media — Adapted INCOM | Within-subject variation (no between-group comparator) | Depression | Daily diary (14-day) | 1 |
| 19 | Liu et al. (2023) | Adolescent | China (Eastern) | 927 | 2023 | Upward social comparison on social media — Adapted Gibbons & Buunk social comparison scale | Within-subject variation (no between-group comparator) | Self-Esteem | Cross-sectional | 1 |
| 20 | Zhang et al. (2025) | Adult | China (Eastern) | 789 | 2023 | Upward social comparison on social media — Online upward social comparison scale (Liang et al. revision of Gibbons & Buunk) | Within-subject variation (no between-group comparator) | Anxiety; Self-Esteem | Cross-sectional | 2 |
| 21 | Lee (2022) | Adult | South Korea (Eastern) | 236 | 2019 | Upward social comparison on social media — Adapted social comparison orientation scale | Within-subject variation (no between-group comparator) | Well-Being | Cross-sectional | 1 |
| 22 | Tian et al. (2025) | Adult | China (Eastern) | 397 | 2023 | Upward social comparison on social media — Upward social comparison scale | Within-subject variation (no between-group comparator) | Anxiety | Cross-sectional | 1 |
| 23 | Li (2018) | Adolescent | China (Eastern) | 934 | 2018 | Upward social comparison on social media — Upward social comparison scale (Chinese) | Within-subject variation (no between-group comparator) | Depression; Social-Evaluative Negative Emotions | Cross-sectional | 2 |
| 24 | Park et al. (2021) | Adult | South Korea (Eastern) | 330 | 2019 | Upward social comparison on social media — Upward comparison scale | Within-subject variation (no between-group comparator) | Well-Being | Cross-sectional | 1 |
| 25 | Li et al. (2025) | Adult | China (Eastern) | 200 | 2025 | Upward social comparison on social media — Self-reported emotional response measure | Within-subject variation (no between-group comparator) | Social-Evaluative Negative Emotions; Self-Esteem | Cross-sectional | 2 |
| 26 | Lim et al. (2019) | Adult | South Korea (Eastern) | 552 | 2018 | Upward social comparison on social media — Upward comparison scale | Control / no-exposure condition | Social-Evaluative Negative Emotions; Well-Being | Quasi-experimental | 4 |
| 27 | Zheng et al. (2020) | Adolescent | China (Eastern) | 799 | 2018 | Upward social comparison on social media — INCOM upward comparison subscale | Within-subject variation (no between-group comparator) | Anxiety | Cross-sectional | 1 |
| 28 | Wang et al. (2020) | Adult | China (Eastern) | 514 | 2018 | Upward social comparison on social media — Adapted INCOM (mobile version) | Within-subject variation (no between-group comparator) | Depression; Social-Evaluative Negative Emotions | Cross-sectional | 2 |
| 29 | Li et al. (2024) | Adult | China (Eastern) | 329 | 2023 | Upward social comparison on social media — INCOM upward comparison subscale (online version) | Within-subject variation (no between-group comparator) | Anxiety | Cross-sectional | 1 |
| 30 | Liu et al. (2017) | Adult | China (Eastern) | 1205 | 2016 | Upward social comparison on social media — Upward social comparison scale (Chinese revised version) | Within-subject variation (no between-group comparator) | Depression; Self-Esteem | Cross-sectional | 2 |
| 31 | Yang et al. (2023) | Adult | China (Eastern) | 462 | 2022 | Upward social comparison on social media — Iowa–Netherlands social comparison scale | Within-subject variation (no between-group comparator) | Anxiety; Self-Esteem | Cross-sectional | 2 |
| 32 | Jeong & Hyun (2017) | Adult | South Korea (Eastern) | 186 | 2017 | Upward social comparison on social media — Instagram-based comparison experience measure | Within-subject variation (no between-group comparator) | Depression | Cross-sectional | 1 |
| 33 | Lian et al. (2017) | Adolescent | China (Eastern) | 836 | 2015 | Upward social comparison on social media — INCOM comparison subscale | Within-subject variation (no between-group comparator) | Depression; Social-Evaluative Negative Emotions | Cross-sectional | 2 |
| 34 | Frison & Eggermont (S1) (2016) | Adolescent | Western | 1621 | 2014 | Upward social comparison on social media — Self-reported negative comparison on Facebook | Within-subject variation (no between-group comparator) | Well-Being | Longitudinal | 1 |
| 35 | Frison & Eggermont (S2) (2016) | Adolescent | Western | 1621 | 2014 | Upward social comparison on social media — Self-reported negative comparison on Facebook | Within-subject variation (no between-group comparator) | Well-Being | Longitudinal | 1 |
| 36 | Schmuck et al. (S1) (2019) | Adult | Western | 833 | 2017 | Upward social comparison on social media — Mobile SNS upward comparison measure | Within-subject variation (no between-group comparator) | Self-Esteem; Well-Being | Longitudinal | 2 |
| 37 | Schmuck et al. (S2) (2019) | Adult | Western | 461 | 2017 | Upward social comparison on social media — Mobile SNS upward comparison measure | Within-subject variation (no between-group comparator) | Self-Esteem; Well-Being | Longitudinal | 2 |
| 38 | Xiang et al. (2024) | Adolescent | China (Eastern) | 744 | 2024 | Upward social comparison on social media — Upward social comparison scale (Lian et al., 2017) | Within-subject variation (no between-group comparator) | Self-Esteem | Cross-sectional | 1 |
| 39 | Pang (2021) | Adult | China (Eastern) | 318 | 2019 | Upward social comparison on social media — Upward social comparison items (adapted from De Vries & Kühne, 2015) | Within-subject variation (no between-group comparator) | Social-Evaluative Negative Emotions | Cross-sectional | 1 |
| 40 | Yuan et al. (S1) (2025) | Adolescent | China (Eastern) | 1179 | 2025 | Upward social comparison on social media — Upward social comparison subscale (Gibbons & Buunk, 1999) | Within-subject variation (no between-group comparator) | Depression; Self-Esteem | Longitudinal | 2 |
| 41 | Yuan et al. (S2) (2025) | Adolescent | China (Eastern) | 1179 | 2025 | Upward social comparison on social media — Upward social comparison subscale (Gibbons & Buunk, 1999) | Within-subject variation (no between-group comparator) | Depression; Self-Esteem | Longitudinal | 2 |
| 42 | Niu et al. (2016) | Adolescent | China (Eastern) | 964 | 2015 | Upward social comparison on social media — Upward social comparison questionnaire | Within-subject variation (no between-group comparator) | Depression; Self-Esteem | Cross-sectional | 2 |
| 43 | Li (2024) | Adult | China (Eastern) | 1078 | 2023 | Upward social comparison on social media — Upward Social Comparison Scale | Within-subject variation (no between-group comparator) | Well-Being; Social-Evaluative Negative Emotions | Cross-sectional | 2 |
| 44 | Tong et al. (2017) | Adolescent | China (Eastern) | 836 | 2016 | Upward social comparison on social media — INCOM upward comparison subscale | Within-subject variation (no between-group comparator) | Anxiety; Depression | Cross-sectional | 2 |
| 45 | Sun et al. (2016) | Adolescent | China (Eastern) | 603 | 2015 | Upward social comparison on social media — INCOM upward comparison subscale | Within-subject variation (no between-group comparator) | Depression | Cross-sectional | 1 |
| 46 | Wang et al. (2017) | Adult | Eastern | 696 | 2016 | Upward social comparison on social media — Negative upward social comparison affect scale (2 items) | Within-subject variation (no between-group comparator) | Self-Esteem; Well-Being | Cross-sectional | 2 |
| 47 | Li & Wu (2025) | Adult | China (Eastern) | 562 | 2024 | Upward social comparison on social media — Upward comparator behaviour scale / INCOM | Within-subject variation (no between-group comparator) | Anxiety | Cross-sectional | 1 |
| 48 | Park & Park (2024) | Adult | Eastern | 432 | 2023 | Upward social comparison on social media — Negative upward comparison scale (3 items, based on Lim & Yang, 2015) | Within-subject variation (no between-group comparator) | Social-Evaluative Negative Emotions | Cross-sectional | 1 |
| 49 | Gao et al. (2024) | Adult | Eastern | 616 | 2023 | Upward social comparison on social media — Upward social comparison on SNSs scale (6 items) | Within-subject variation (no between-group comparator) | Social-Evaluative Negative Emotions | Cross-sectional | 1 |
| 50 | Ling et al. (2023) | Adult | Eastern | 568 | 2022 | Upward social comparison on social media — Upward social comparison on SNS scale | Within-subject variation (no between-group comparator) | Social-Evaluative Negative Emotions | Cross-sectional | 1 |
| 51 | Ladwig et al. (2025) | Adult | Western | 131 | 2024 | Upward social comparison on social media — Upward Photographic Appearance Comparison Scale (UPACS) | Control / no-exposure condition | Social-Evaluative Negative Emotions | Experimental (fitspiration exposure) | 1 |
| 52 | Le Blanc-Brillon et al. (Study 1) (2025) | Adult | Canada/USA (Western) | 139 | 2021 | Upward social comparison on social media — Vogel et al. (2014) Social comparisons questionnaire (Instagram upward comparison item) | Within-subject variation (no between-group comparator) | Self-Esteem | Cross-sectional | 2 |
| 53 | Le Blanc-Brillon et al. (Study 2) (2025) | Adult | Western | 413 total (Facebook n=207; Instagram n=206) | 2023 | Upward social comparison on social media — Vogel et al. (2015) perceived upward comparison items; platform-specific correlations extracted separately for Facebook and Instagram subsamples | Within-subject variation (no between-group comparator) | Depression; Self-Esteem | Cross-sectional | 6 |
| 54 | Robinson et al. (2019) | Adult | USA (Western) | 504 | 2018 | Upward social comparison on social media — single-item upward comparison (“people better off than you”) | MDD vs. no-MDD comparison groups | Depression | Cross-sectional | 1 |

**Notes.** k = number of effect sizes contributed by the study. Population: Adolescent = mean age < 18 years; Adult = mean age ≥ 18 years. Cultural Background: coded as Western or Eastern based on the country in which data were collected. Outcomes: Anxiety; Depression; Well-Being (subjective well-being / life satisfaction / happiness); Self-Esteem (global self-esteem / self-worth); Social-Evaluative Negative Emotions (Social-Evaluative Negative Emotions, including envy, body dissatisfaction, fear of negative evaluation, and loneliness). Comparator: For correlational and longitudinal studies, no between-group comparator was applicable; associations were estimated within-sample. For experimental studies, the comparator refers to a control or low-exposure condition. Studies labeled (S1) and (S2) denote independent subsamples reported within the same article and treated as separate units of analysis in the three-level model.
