# Supplementary figures and images for: “Looking up” linked to feeling down: a meta-analysis of online upward social comparison and psychological maladjustment
Source: Front Psychol. 2026 May 15;17:1825169. doi: 10.3389/fpsyg.2026.1825169 (PMC13219356; doi:10.3389/fpsyg.2026.1825169)

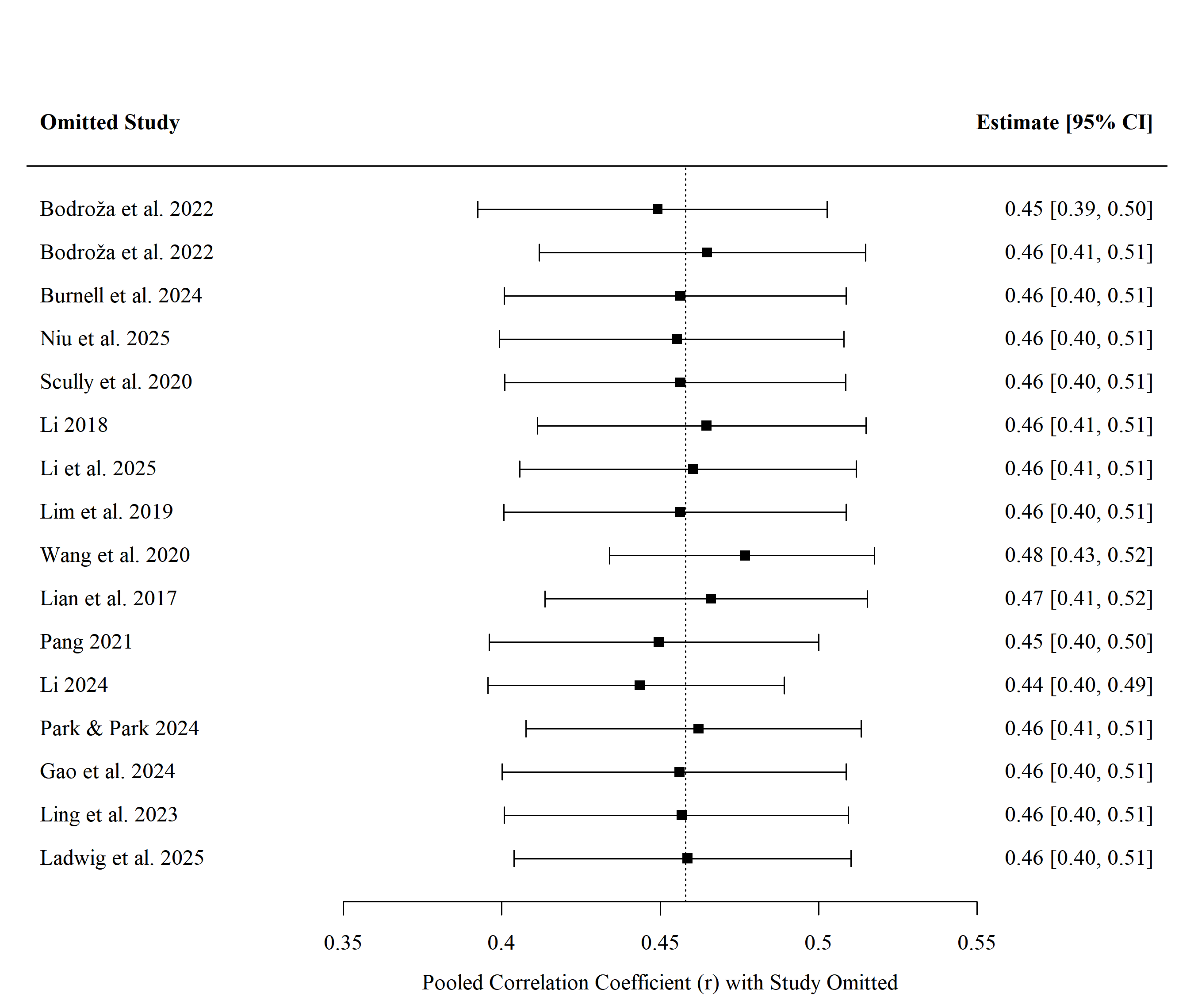

Supplement: Supplementary file 1 [file Data_Sheet_1.zip › Supplementary Material/SF10_LOO_Social-Evaluative_Negative_Emotions.tiff]

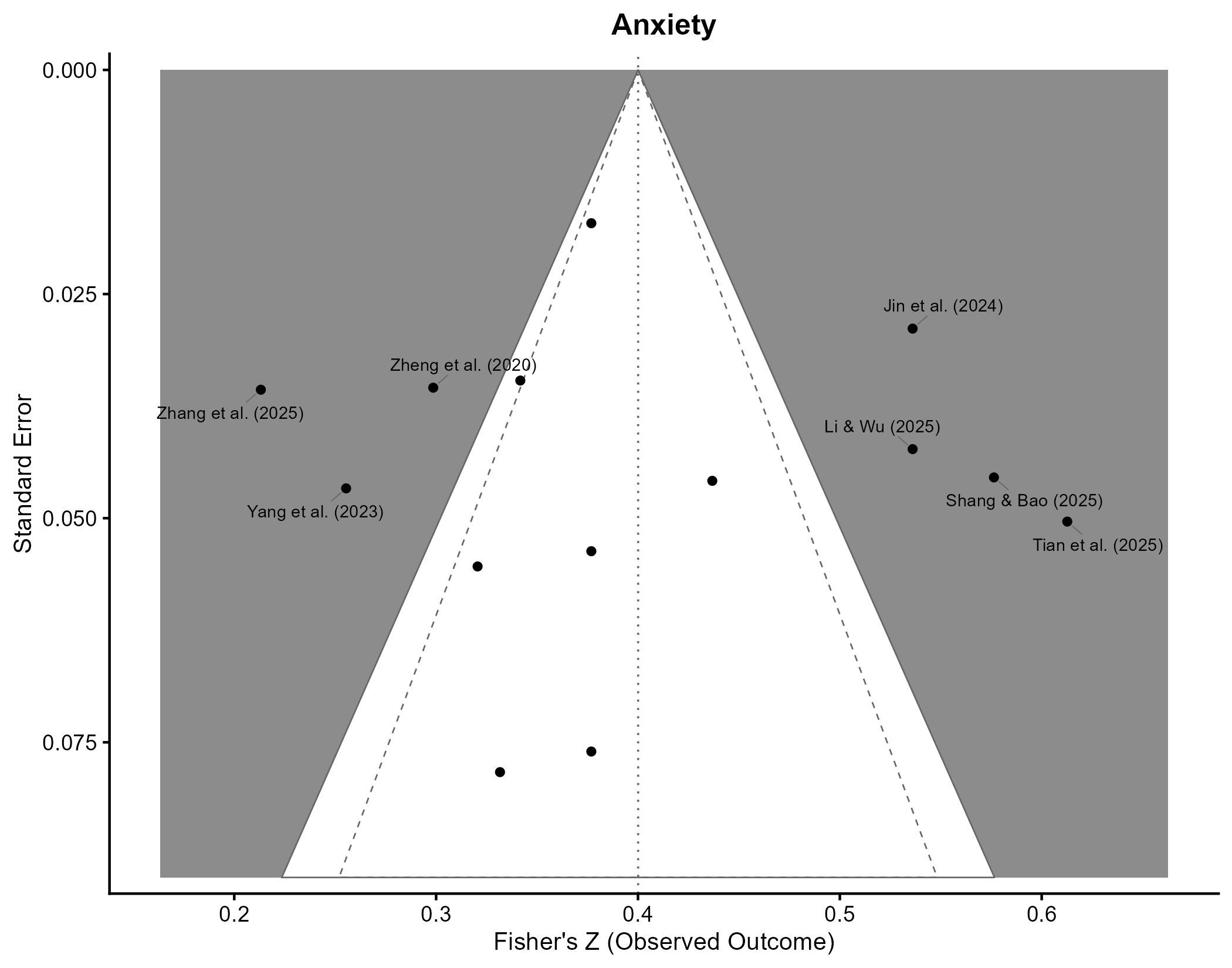

Supplement: Supplementary file 1 [file Data_Sheet_1.zip › Supplementary Material/SF1_Funnel_Anxiety.tiff]

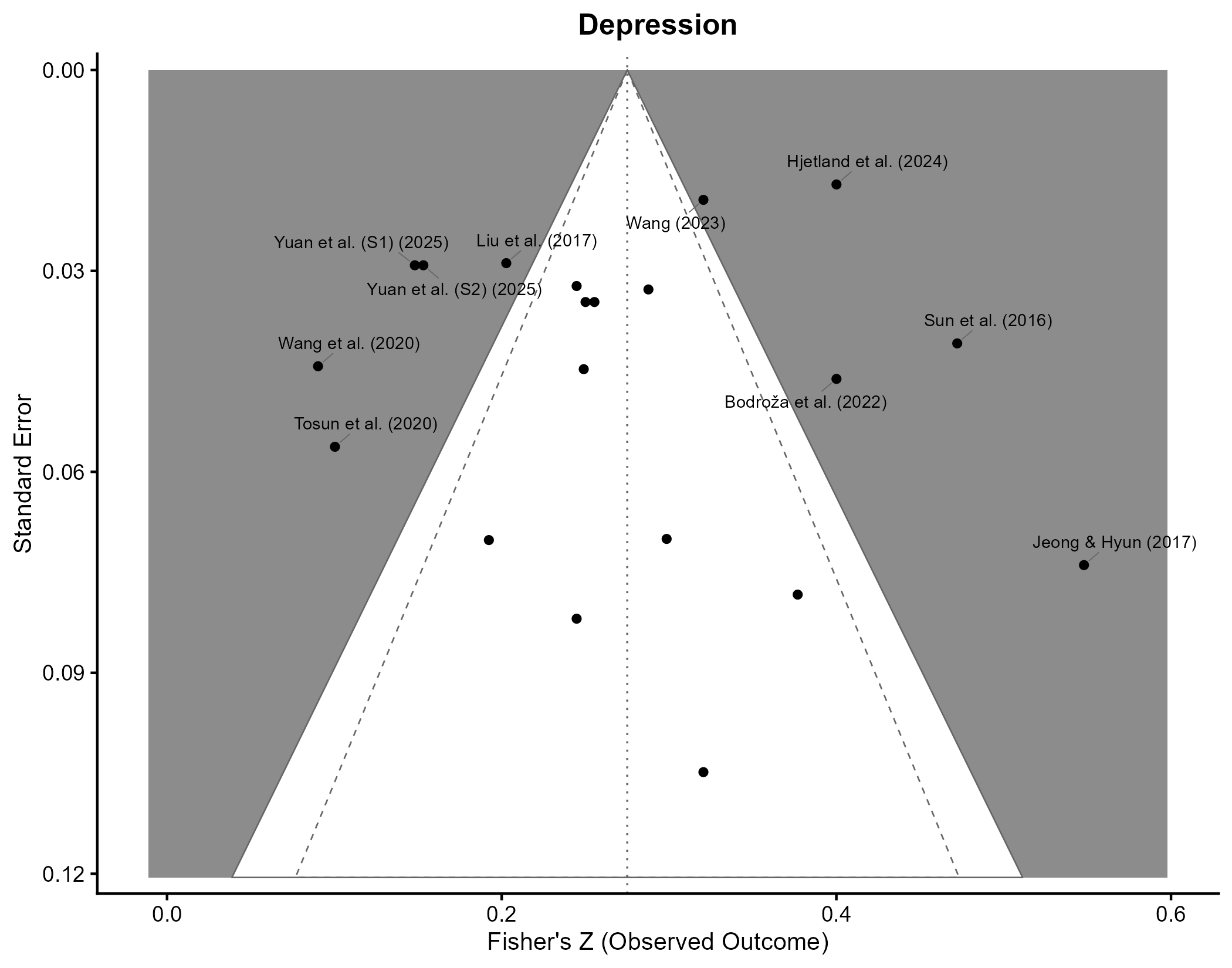

Supplement: Supplementary file 1 [file Data_Sheet_1.zip › Supplementary Material/SF2_Funnel_Depression.tiff]

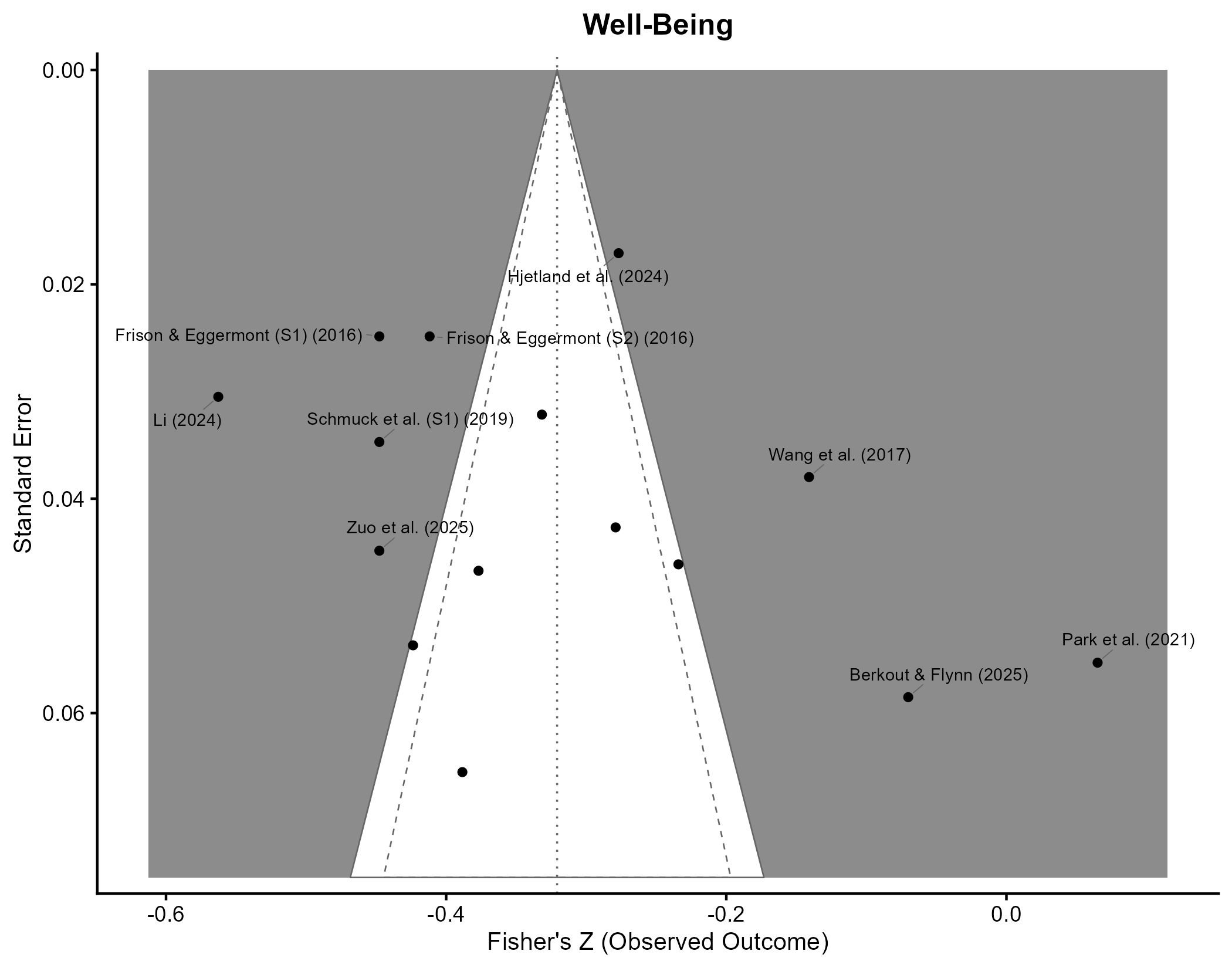

Supplement: Supplementary file 1 [file Data_Sheet_1.zip › Supplementary Material/SF3_Funnel_Well_Being.tiff]

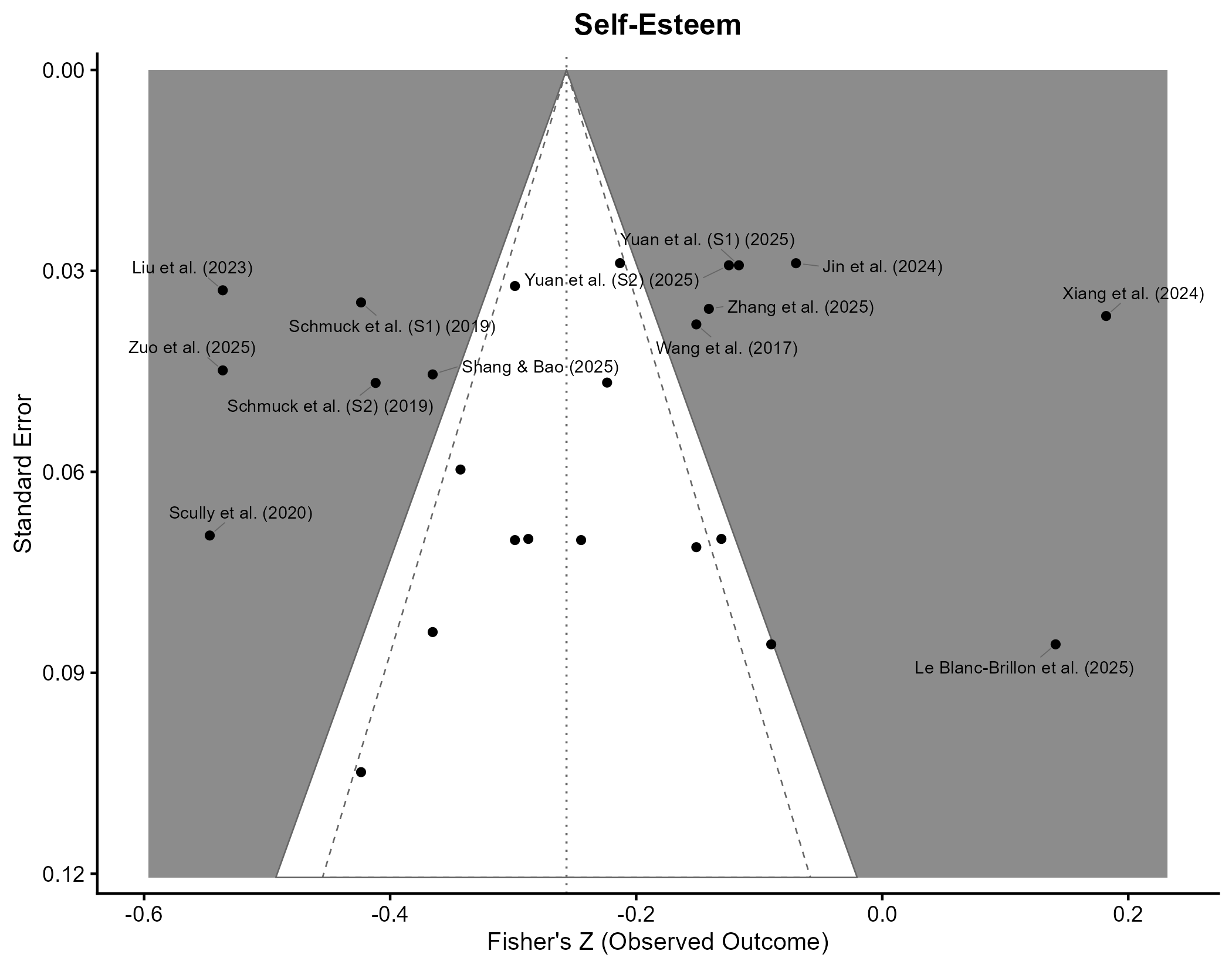

Supplement: Supplementary file 1 [file Data_Sheet_1.zip › Supplementary Material/SF4_Funnel_Self_Esteem.tiff]

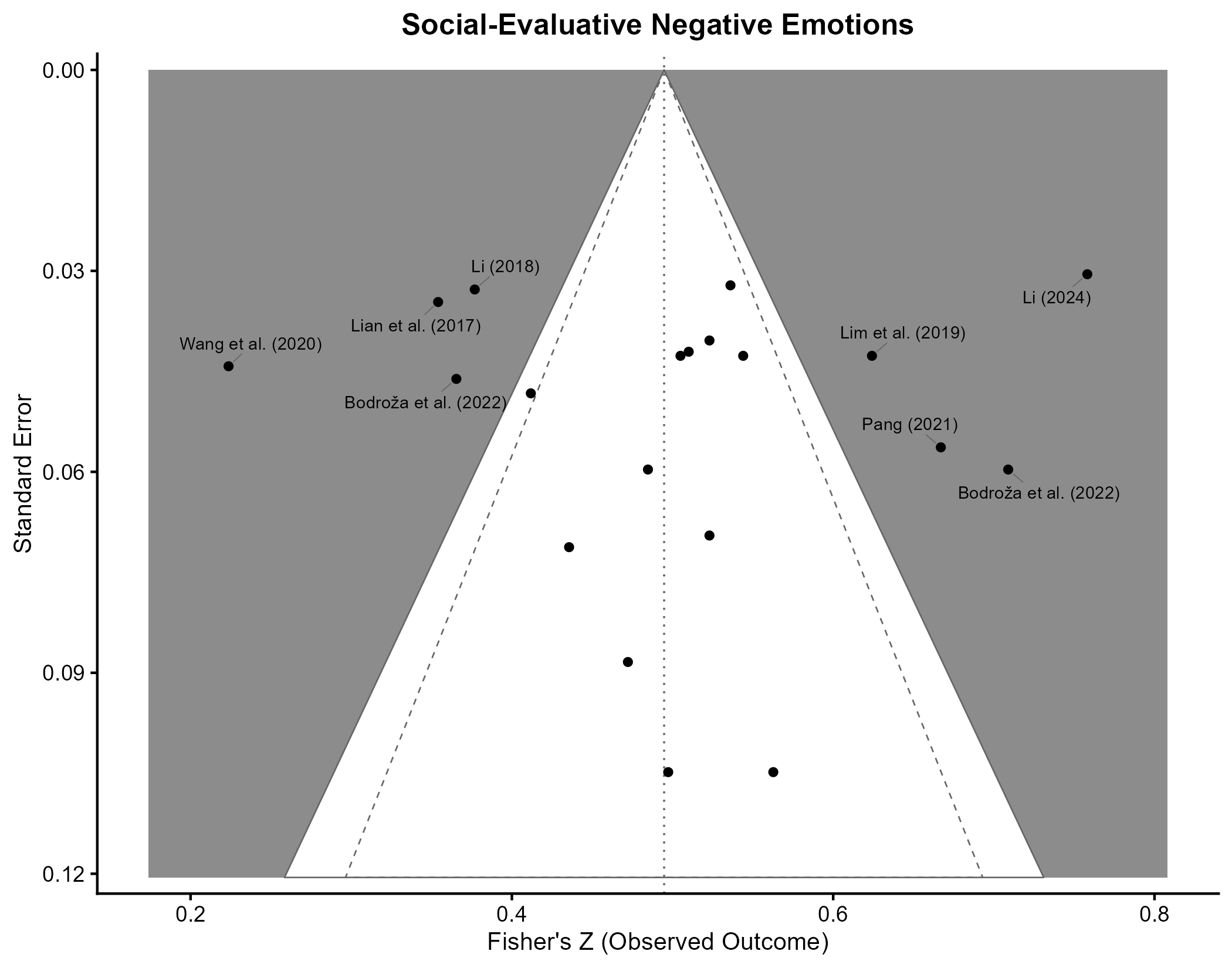

Supplement: Supplementary file 1 [file Data_Sheet_1.zip › Supplementary Material/SF5_Funnel_Social-Evaluative_Negative_Emotions.tiff]

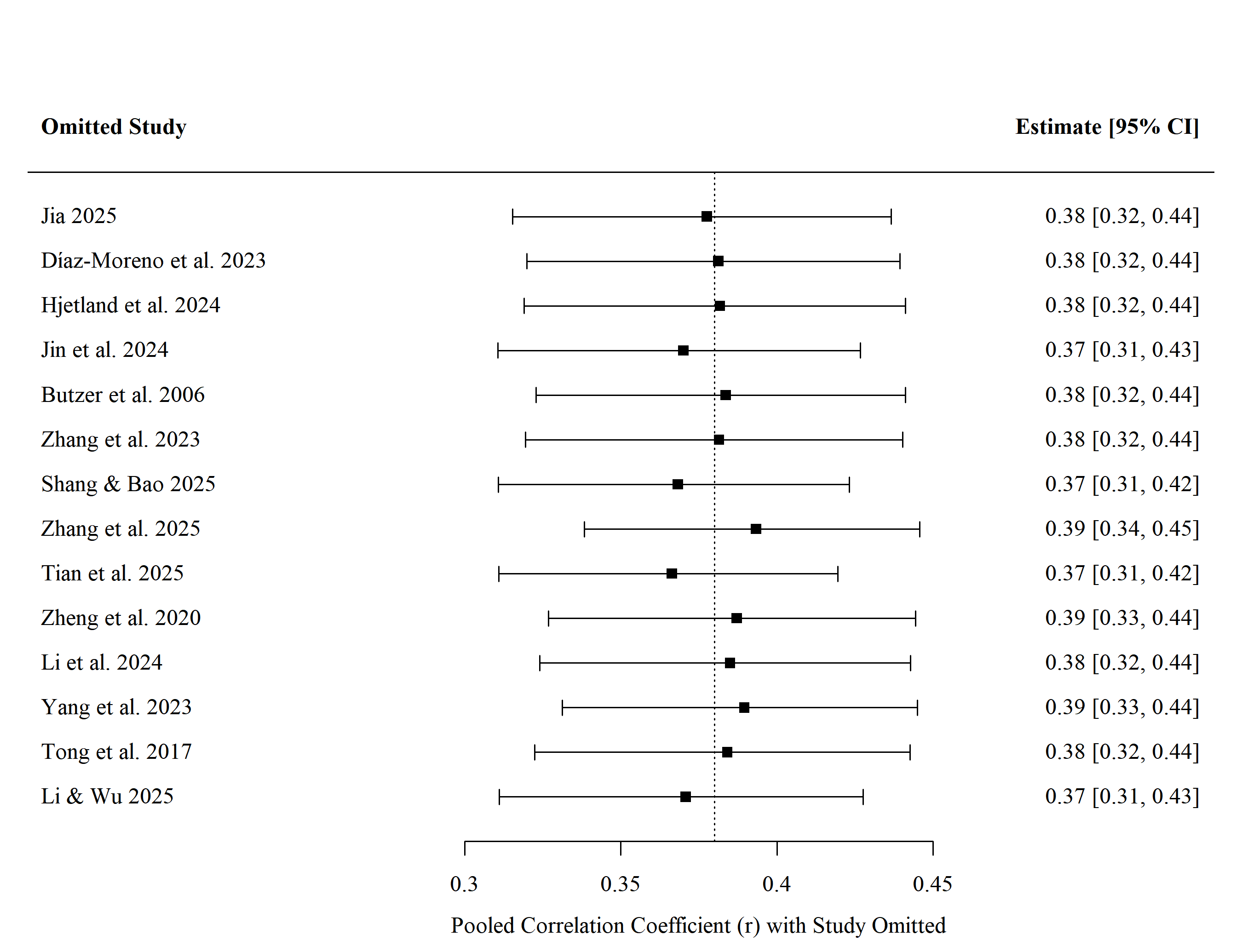

Supplement: Supplementary file 1 [file Data_Sheet_1.zip › Supplementary Material/SF6_LOO_Anxiety.tiff]

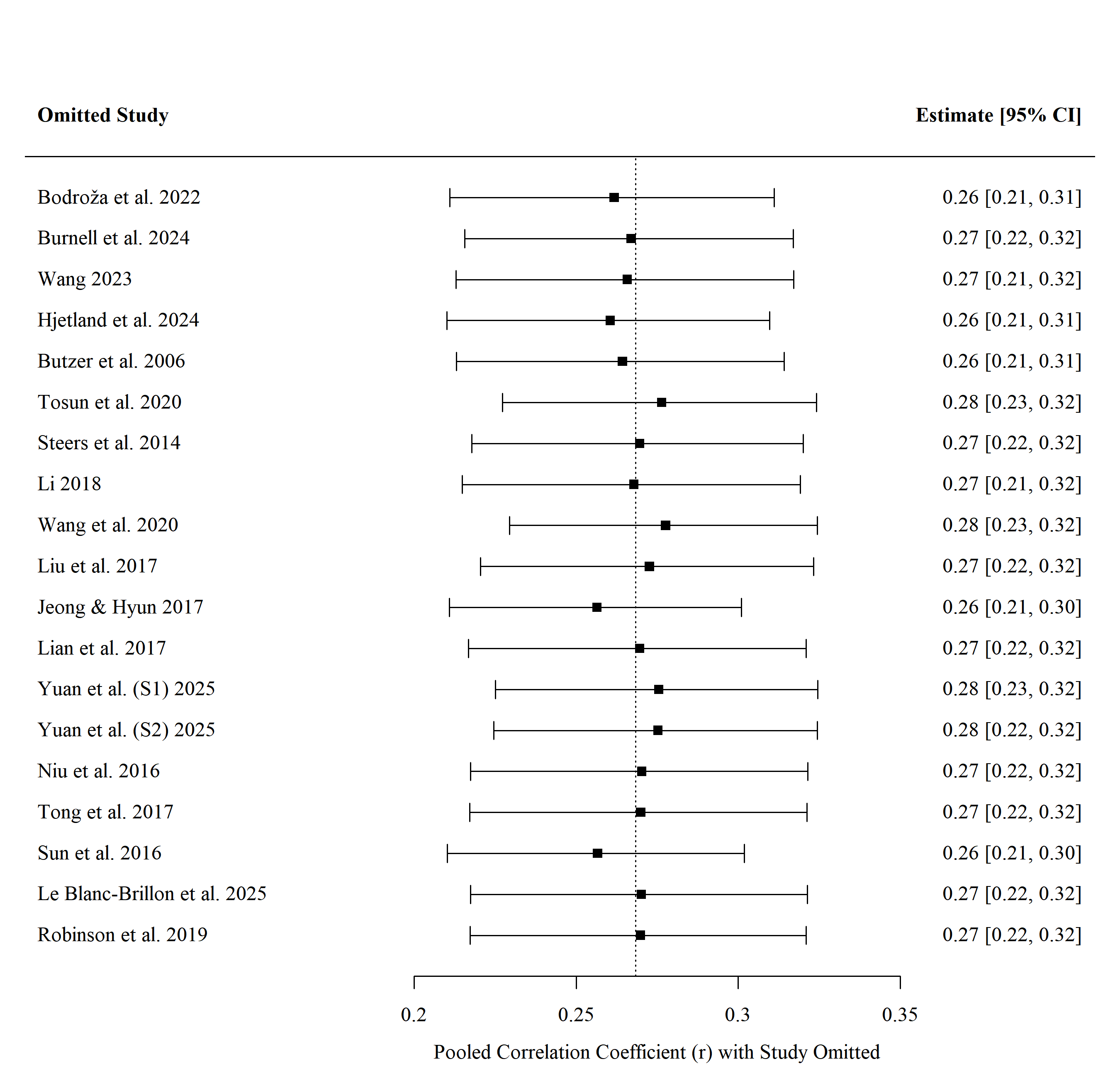

Supplement: Supplementary file 1 [file Data_Sheet_1.zip › Supplementary Material/SF7_LOO_Depression.tiff]

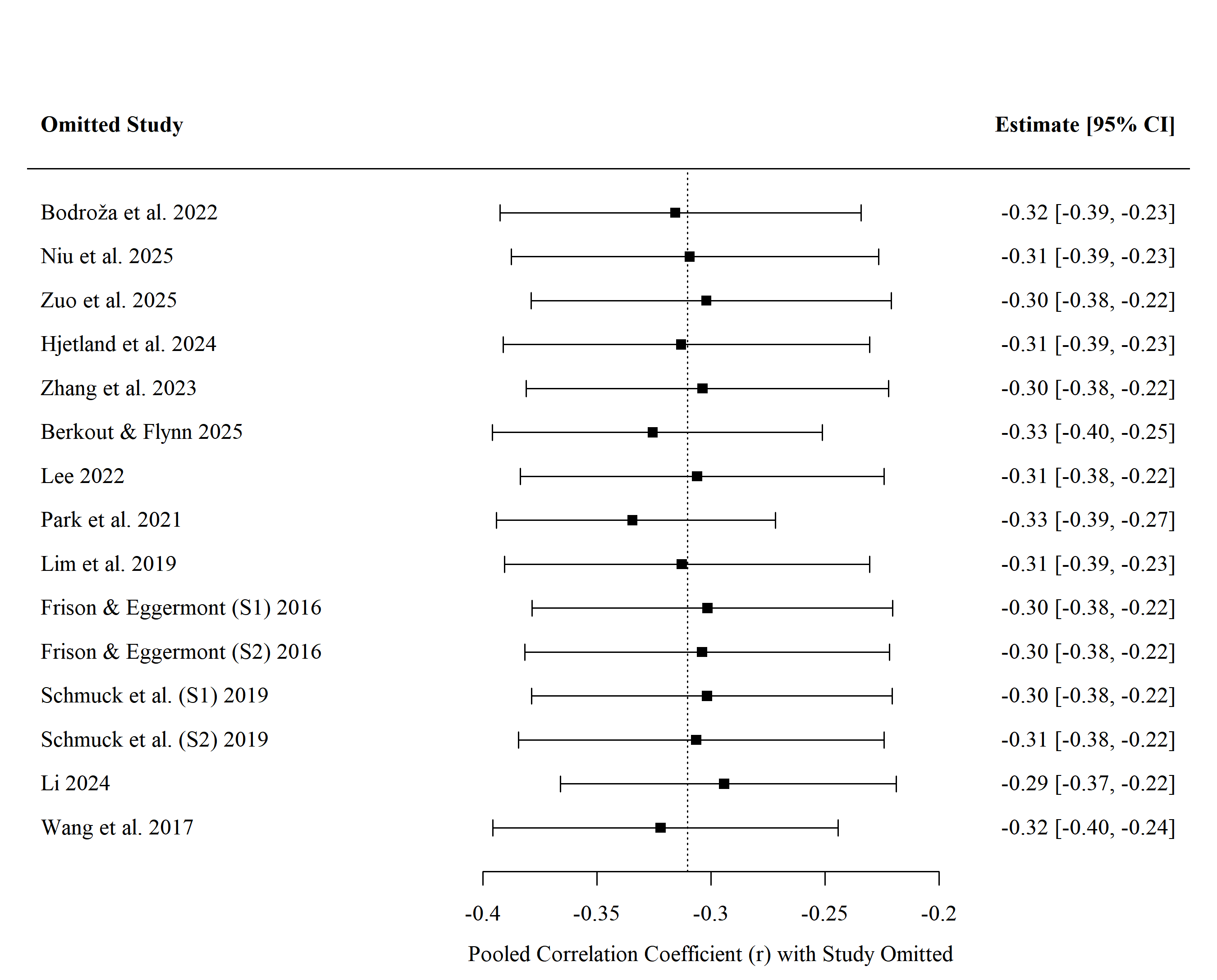

Supplement: Supplementary file 1 [file Data_Sheet_1.zip › Supplementary Material/SF8_LOO_Well_Being.tiff]

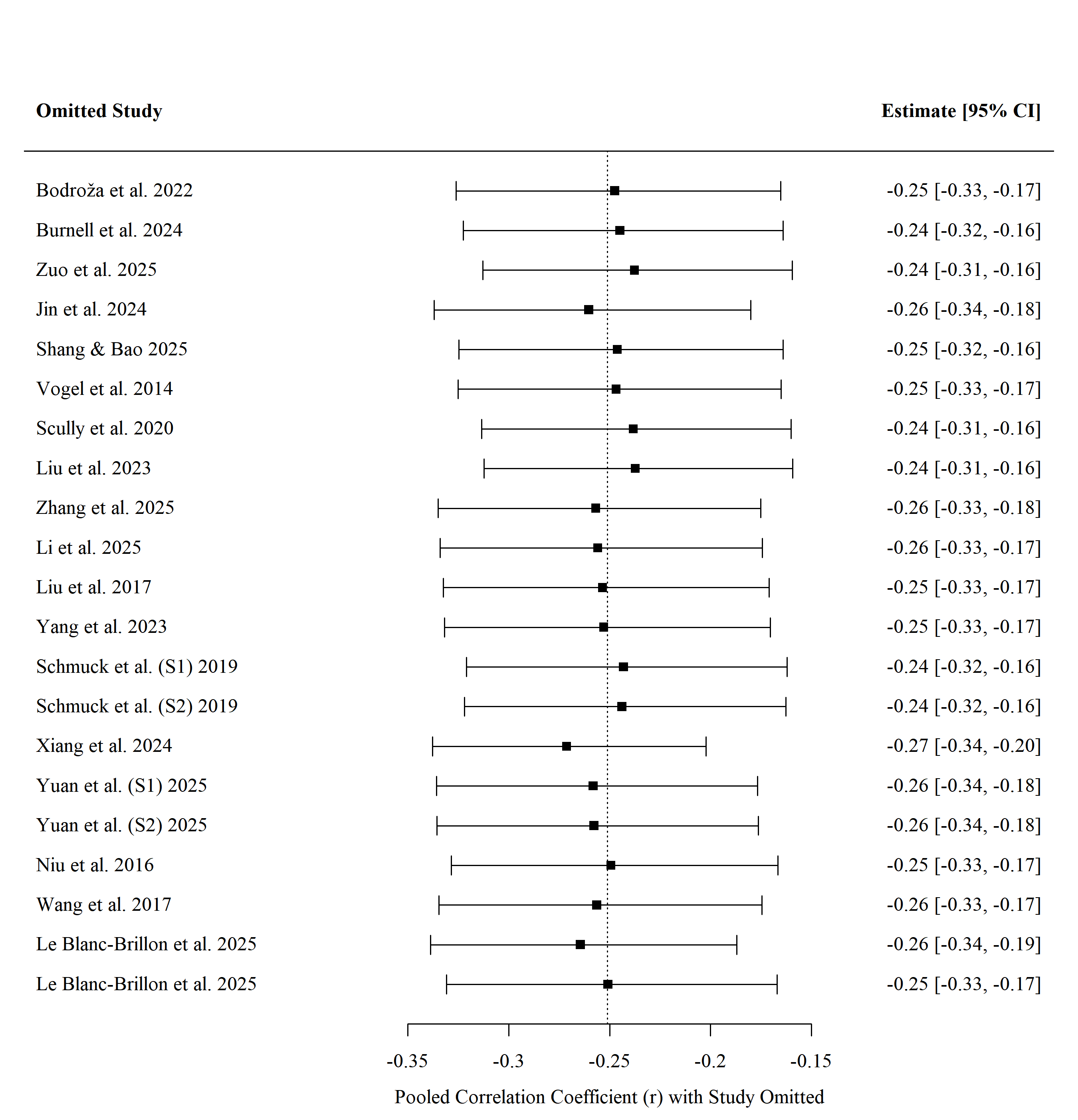

Supplement: Supplementary file 1 [file Data_Sheet_1.zip › Supplementary Material/SF9_LOO_Self_Esteem.tiff]
